# An Evaluation of Different NIR-Spectral Pre-Treatments to Derive the Soil Parameters C and N of a Humus-Clay-Rich Soil

**DOI:** 10.3390/s21041423

**Published:** 2021-02-18

**Authors:** Kurt Heil, Urs Schmidhalter

**Affiliations:** 1 Chair of Plant Nutrition, Technical University Munich, Emil-Ramann-Str. 2, D-85350 Freising, Germany; schmidhalter@wzw.tum.de; 2 Chair of Agricultural Systems Engineering, Technical University Munich, Dürnast 4, D-85354 Freising, Germany

**Keywords:** near-infrared spectroscopy (NIRS), partial least squares regression (PLSR), pre-treatments, soil properties (C, N), soil heterogeneity

## Abstract

Near-infrared reflectance spectroscopy (NIRS) was successfully used in this study to measure soil properties, mainly C and N, requiring spectral pre-treatments. Calculations in this evaluation were carried out using multivariate statistical procedures with preceding pre-treatment procedures of the spectral data. Such transformations could remove noise, highlight features, and extract essential wavelengths for quantitative predictions. This frequently significantly improved the predictions. Since selecting the appropriate transformation was not straightforward due to the large numbers of available methods, more comprehensive insight into choosing appropriate and optimized pre-treatments was required. Therefore, the objectives of this study were (i) to compare various pre-processing transformations of spectral data to determine their suitability for modeling soil C and N using NIR spectra (55 pre-treatment procedures were tested), and (ii) to determine which wavelengths were most important for the prediction of C and N. The investigations were carried out on an arable field in South Germany with a soil type of Calcaric Fluvic Relictigleyic Phaeozem (Epigeoabruptic and Pantoclayic), created in the flooding area of the Isar River. The best fit and highest model accuracy for the C (Ct, Corg, and Ccarb) and N models in the calibration and validation modes were achieved using derivations with Savitzky–Golay (SG). This enabled us to calculate the Ct, Corg, and N with an R^2^ higher than 0.98/0.86 and an ratio of performance to the interquartile range (RPIQ) higher than 10.9/4.1 (calibration/validation).

## 1. Introduction

Near-infrared reflectance spectroscopy (NIRS) has been used successfully to characterize many soil properties, including C, N, cation exchange capacity, and soil texture. In comparison to conventional laboratory methods, NIRS is inexpensive, fast, non-destructive, produces no chemical substances, and requires minimal sample preparation, making it highly attractive. Several soil properties can also be determined with a single scan. Some researchers have noted that this technique can estimate primary soil properties (such as total C, N, and exchange capacity), as well as secondary soil properties (such as the respiration rate and potentially mineralizable N) at the same time [1,2,3,4,5,6]. Other authors have described the application of additional physical and soil chemical properties [7,8,9,10,11,12].

Near-infrared spectroscopy measures the reflection of radiation with wavelengths from 750 to 2500 nm. The NIR spectra contain broad bands that are produced by reflections with overlapping wavelengths. Reflections measured by this type of spectroscopy mainly correspond to combinations of vibrational modes with chemical C–H, O–H, overtones, and N–H bonds [13].

The recorded reflectance produces spectra that are unique for this sample. The collected reflectance lines include data on the molecules’ properties in the sample and provide essential information about the sample composition.

However, the raw spectra also contain some information that complicates the evaluation. For example, background interference, overlapping absorption bands from other constituents, noise (caused by matrix/environmental effects, water absorption bands, instrumental noise, extraneous light sources), and weak deflections can make the mathematical derivations between light and particles more difficult or sometimes even impossible.

Therefore, it is common practice to carry out pre-processing and special calibration/validation procedures to reduce such effects and obtain valuable information about the soil samples’ properties.

According to Gholizadeh et al. [4,5,6], the main challenge here is limiting the application of NIRS for the evaluation of soil properties. Finding suitable pre-treatments and identifying calibration/validation strategies is also complicated because there are many techniques available. The high number of methods renders an overview difficult.

Although many pre-processing transformations have been used in NIRS, the choice between them is somewhat arbitrary, and little is known about how this choice affects the final prediction of soil properties [14,15]. Indeed, finding an appropriate method needs more time than the main calculations. Table 1 gives an overview of the main applied pre-processing techniques used in the last ten years.

According to Gholizade et al. [4,5,6] and Xie et al. [16], these techniques can be divided into four main groups: Scaling, smoothing, baseline correction, normalization. Dotto et al. [17] grouped these methods into only two categories: Scatter correction and spectral derivation. Scatter corrections are represented by continuum distance, normalization by range, standard normal variables, and multiplicative dispersion correction. The pre-processing of spectral derivatives includes Savitzky–Golay and Norris–Williams derivatives [17]. In the following section, a basic overview of these procedures is given.

The smoothing procedure is used to reduce noise and is also referred to as a measurement error. Commonly used smoothing methods include the moving average, median filters, and Savitzky–Golay transformation [18]. In current soil-related studies, the moving average is the dominant smoothing practice.

A scaling procedure is applicable when the intensity of different spectra needs to be scaled so that the specifications can be compared. Scaling produces a spectrum with a mean of zero and a standard deviation of 1. Here, the procedure corrects light scattering by centering and scaling each spectrum.

Using baseline removal, background signals that are far from the zero lines are removed [4,5,6,19,20]. These signals must be removed, as they will cause peak heights and peak areas to be incorrectly calculated. The methods here are derivatives and are some of the best methods for eliminating baseline effects. According to Gholizade et al. [3,5,6], a different method to eliminate scattering is a multiplicative signal correction (MSC), a transformation method applied to equalize multiplicative and/or additive scattering effects [16].

Normalization generates new spectral data via the creation of shifted and scaled data. With these normalized values, scattering effects (e.g., changing slopes) are eliminated.

Some soil parameters are frequently derived with vis-NIR spectroscopy. The most apparent properties are soil organic carbon (SOC), total soil carbon, soil organic matter (humus content), and clay. However, achievements of the quality of carbon are rare. The applied methods for deriving these and other soil properties are listed in Vasques et al. [14,15]. Mainly referring to the last decade of research, Table 1 outlines the target variables and the used pre-processing techniques.

This table provides the results from a query of the ResearchGate database to select publications featuring the application of pre-processing methods to predict soil properties over the last ten years.

Within this catalogue of transformations, some methods were more frequently used than others, including smoothing with different search windows, normalization, standardization, and derivation with Savitzky–Golay [14,15,19,23,24,25,27,28,30,31,32,35,36].

The selection of multivariate techniques was similarly difficult. Upon the first examination, regressions seemed to be an appropriate statistical method for building reliable calibration models. However, the multicollinearity of highly correlated wavelengths required additional methods. These methods were mainly principal component regression (PCR) and partial least squares regression (PLSR). Instead of using the original data, these methods develop orthogonal (uncorrelated) linear combinations from the spectral variables (components or factors). Other methods have also been established for analyzing spectrometer readings, including support vector machine (SVM), soft independent modeling of a class anthology (SIMCA), cluster analysis (CA), discriminant partial least square (DPLS), K-nearest neighbors (KNN), and linear discriminant analysis (LDA). A comprehensive review of the relevant qualitative methods and their applications can be found in Cen and He [37].

The derivation of a calibration model also involves validations. A commonly used validation method is internal or cross-validation, where the validation dataset is a part of the calibration dataset. In contrast, external validations use an independent data sample set (test set) and provide more reliable and relevant estimates. In general, in both procedures, it is necessary to select a representative sample set that provides the most considerable amount of information for the calculation, including as many variations as possible that may occur in future samples [20].

Among all multivariate statistics, PLSR has been applied the most frequently. Dotto et al. [17] searched the scientific citation database Scopus and found that from 2006 to 2016, publications with PLSR appeared with a frequency of around 65% for predicting soil properties with NIRS. This is the reason why this procedure is applied in the present evaluation.

The soil investigated in this work occupies a particular position among the various soils investigated, and its high clay content in combination with high C-content has not yet been described in the literature. The most frequently examined soil types are Ultisol, Spodosol, Entisol, Cambisol, Luvisol, Oxisol, and Alfisol, as shown in Table 1.

Therefore, the specific objectives were the following: (i) To compare various pre-processing transformations of spectral data to determine their suitability for modeling C (Ct, Corg, Ccarb) and N using NIR spectra with PLSR, and (ii) to evaluate which wavelengths are most important for the prediction of the selected parameters.

## 2. Materials and Methods

### 2.1. General Description, Soil, and Physiography

The study area is approximately 1.0 ha in size and is located in Freising, 30 km north of Munich, Germany (44°78′977″ E, 535°87′77″ N), in a formerly flooded area of the Isar River.

**Figure 1:**
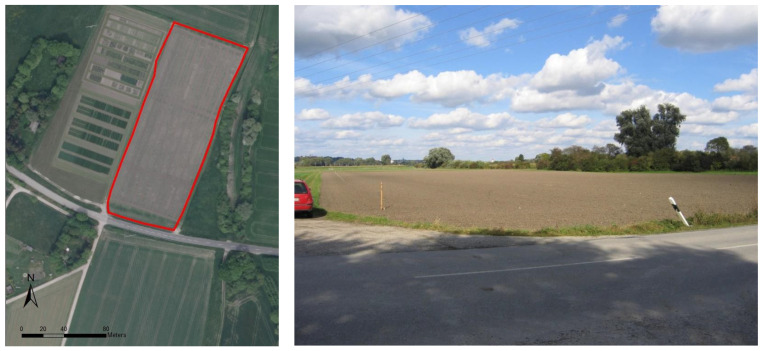
Aerial image and picture of the position of the field (Pulling 8) in the landscape.

**Figure 2:**
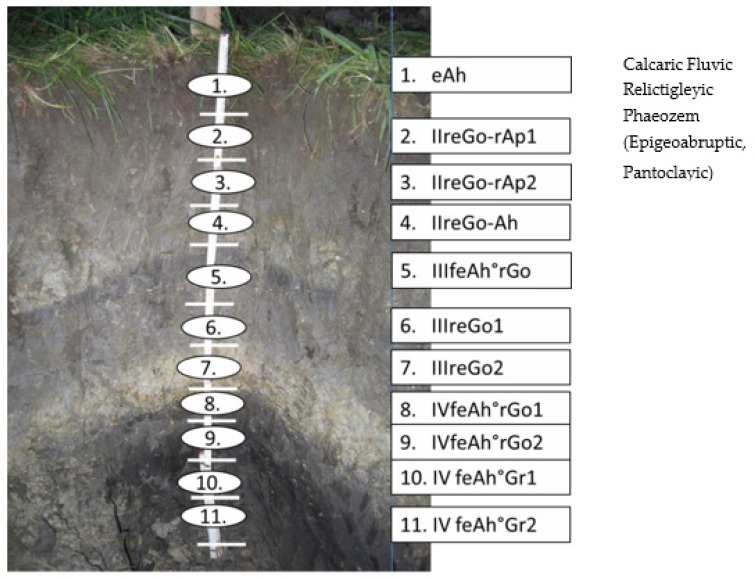
The soil profile of the investigated field Pulling 8 with a description of the names of the horizons and vertical discontinuities (abbreviations from [38]).

**Figure 3:**
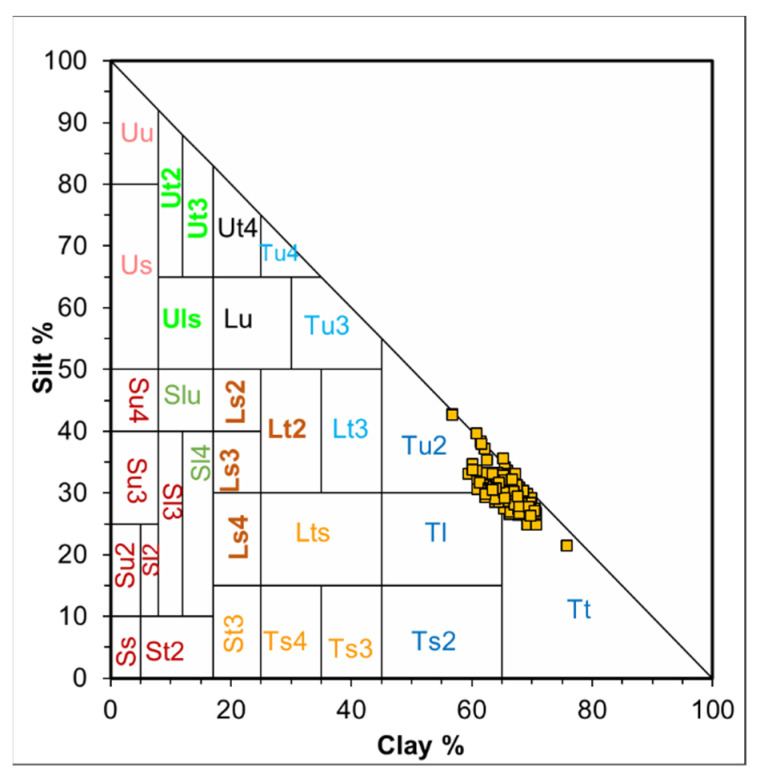
The particle size distribution of the whole soil profile Pulling 8.

The average annual temperature in the area is approximately 7.8 °C, and the average annual precipitation is 800 mm. Holocene sediments are the predominant soil material. The composition of this flat area is a consequence of repeated floods with periods of soil-forming processes. According to the German Soil Survey [38] and the WRB, Calcaric Fluvic Relictigleyic Phaeozem is the dominant soil type (see Figure 1, Figure 2 and Figure 3). The soil texture of the field is Tl [38] with loamy clay. The mean clay content is about 65%, with 1% sand. The fine and middle fractions are the dominant texture classes of silt in the area (Table 2).

### 2.2. Soil Sampling and Laboratory Analysis

A total of 120 soil samples were taken from 60 geo-referenced positions down to a depth of 1 m in April 2011 (Figure 1). Soil samples were collected using a regular grid of 10 × 25 m or following the experimental plots’ borders.

Samples were air-dried at room temperature, cleaned of their visible plant residues, and then sieved using a 2-mm mesh sieve.

All samples were subjected to determination of their soil texture (clay, sand siltfine+middle (2–20 µm), siltcoarse (20–63 µm), soil pH, C (Ct, Ccalc, Corg), and N). Soil carbon was analyzed by the dry combustion method using a Costech ECS 4010 Carbon–Nitrogen analyzer (Costech Analytical Technologies Inc., Valencia, CA, USA).

The soil texture was analyzed according to VDLUFA [39].

The soil samples’ spectral reflectance was determined using an FT NIRS Bruker Vector 22/N (Ettlingen, Germany) with a spectral range of 833–2703 nm. The soil samples were distributed homogeneously in glass Petri dishes (9 cm diameter). The constant rotations captured large areas of the sample. The PbS detector scanned an area approximately 2 cm in diameter. A metal stamp (822 g) was used to compress the soil in the dishes to make the sample density uniform and avoid the influence of external light.

During the measurements, a gold standard plate was scanned every 40 min during calibration. Three replications were recorded from each sample. In this way, obviously wrong measurements were identified and eliminated during the measurements. Correct readings were averaged and used for the calculations.

### 2.3. Statistical Data Analysis

#### 2.3.1. Pre-Procession Techniques for Spectral Data

Pre-processing techniques and multivariate statistical methods were applied using the Unscrambler 10.5 [40]. All combinations of pre-processing that were tested and compared are listed in Table 3.

To determine the transformation that delivers the most accurate fit, 55 combinations were tested. All selected transformations are frequently cited in the literature [14,15,22,23,24,25,30,31,32,33,35,36,41].

The compilation of pre-processing procedures in Table 3 is structured into levels that are built upon each other, including smoothing, search window, derivation, and additional techniques.

For smoothing, the readings here were smoothed with different levels. In the first case, the search window was 3, and in the other case, the window was 11 or 25. The derivation techniques of the Savitzky–Golay first derivative, second-order polynomial, Savitzky–Golay second derivative, and second-order polynomial was chosen. The same additional procedure was always selected within each smoothing, search window, and derivation variation (raw data, mean centering, standard normal variate, multiplicative scatter correction, standard normal variate, and standard normal variate and detrending with a second-order polynomial). The first was applied as a control treatment, where the raw reflectance values were not pre-treated.

Such pre-treatment procedures have already been described in the literature [20].

Smoothing was used to reduce background or instrumental noise. The expression of 3 smoothing points indicated 1 point on the right and 1 point on the left of the smoothed point. Eleven smoothing points described 5 points on the left and 5 points on the right of the smoothed reading.

#### 2.3.2. Partial Least Square Regression (PLSR)

After applying the pre-processing techniques, the next step was to build prediction/calibration models with the soil’s physical and chemical properties as dependent target variables.

This PLSR extracted a set of components from the spectra relevant to the target variables [41]. The independent variables were the untreated and pre-treated readings of the wavelengths.

#### 2.3.3. Indices for Evaluation PLSR

Calibration and validation models were assessed to determine their predictive qualities using different indices. Model calibration was performed based on the differences between observed and predicted values after fitting the model using the entire data set. Model validation and the performance of the pre-processing procedures and multivariate statistical methods were evaluated using randomly selected data subsets. For this purpose, the data set was randomly divided into 20 groups, with 15 observations in each group. Ninety percent of the groups were used for model training, and one group was the validation set (prediction set) for model testing. This division in cross-validation and prediction sets was replicated two times.

The applied indices were the coefficient of determination (R^2^), the root mean square error (RMSE), the mean of the difference between the measured and calculated values (Bias), the standard error of prediction (SEP), the ratio of the performance deviation for predicted data (RPDpre), and the ratio of performance deviation for the measured data (RPDlab) [42,43]. R^2^ (the coefficient of determination) is a commonly used measure for the goodness of fit. The RMSE is also commonly used to measure differences between the calculated and observed values from the measured model. The advantage of RMSE is its use of the same data units:

(1) $$R^{2}=\frac{\sum_{i=1}^{n}\hat{(y_{l}}-\bar{y_{l}}{)}^{2}}{\sum_{i=1}^{n}(y_{i}-\bar{y_{l}}{)}^{2}}$$

(2) $$\mathrm{RMSE}=\sqrt{\frac{\sum_{i=t}^{n}{\left({\hat{y}}_{i}-y_{i}\right)}^{2}}{n}}.$$

Bias is the mean difference between the measured and calculated values:

(3) $$\mathrm{Bias}=\frac{y_{i}-{\hat{y}}_{i}}{n}$$

This is defined as the difference between the predicted value and the observed value. A positive value means that the model has overestimated, while a negative value represents an underestimation.

(4) $$\mathrm{SEP}=\mathrm{Standard}\;\mathrm{error}\;\mathrm{of}\;\mathrm{prediction}\;\;\;\mathrm{SEP}=\sqrt{\sum_{i=1}^{n}\frac{y_{i}-{\hat{y}}_{i}}{n}}=\mathrm{SD}*({\hat{y}}_{i}-y_{i}).$$

While the RMSE values indicate the accuracy of the calculation, the SEP index shows the prediction’s precision. SEP squared is approximately equal to the RMSE squared minus the bias squared.

(5) $${\mathrm{RPD}}_{\mathrm{lab}}=\mathrm{ratio}\;\mathrm{of}\;\mathrm{the}\;\mathrm{performance}\;\mathrm{deviation}\;\mathrm{for}\;\mathrm{the}\;\mathrm{measured}\;\mathrm{data}={\mathrm{RPD}}_{\mathrm{lab}}=\frac{{\mathrm{SD}}_{\mathrm{lab}}}{\mathrm{RMSE}}$$

(6) $${\mathrm{RPD}}_{\mathrm{pred}}=\mathrm{ratio}\;\mathrm{of}\;\mathrm{the}\;\mathrm{performance}\;\mathrm{deviation}\;\mathrm{for}\;\mathrm{the}\;\mathrm{predicted}\;\mathrm{data}={\mathrm{RPD}}_{\mathrm{pred}}=\frac{{\mathrm{SD}}_{\mathrm{pred}}}{\mathrm{RMSE}}$$

Here, ȳ is the mean of the observed value, ŷ is the predicted value, y is the observed value, and n is the number of samples with i equal to 1, 2, …, n. SD: Standard deviation observed (lab, measured) and predicted (pred).

While bias, RMSE, and SEP use the same measurement units as the component, the R^2^ and RPD values are dimensionless so that they can be compared similarly between models for different components/properties. Higher values for the RPD indicate more accurate models. The RMSE should be lower than the SD, and the RPD should ideally be four or higher (Table 4). A large RMSE causes low RPD values compared to the SD or the low variability of the reference values.

The ratio of performance to the interquartile range (RPIQ) is a less-often-used index. According to Bellon–Maurel et al. (2010), the soil samples often show skewed distributions instead of normal distributions. The quartiles in the RPIQ better represent the spread of the population.

(7) RPIQ=(Q75–Q25)/RMSE

RPIQ is the difference between the first and third quartiles (Q75–Q25), where Q25 is the value in 25% of the samples, and Q75 is the value in 75% of the samples.

#### 2.3.4. Detection of Important Variables

Variables that have a significant effect are considered essential variables. In addition, the variables involved in important interactions are also important, even if their main effects are negligible.

In such cases, Martens’ automatic uncertainty test indicates the significant variables in the multicomponent model [40].

## 3. Results and Discussion

### 3.1. Characteristics of the Spectral Reflectance Curves

**Figure 4:**
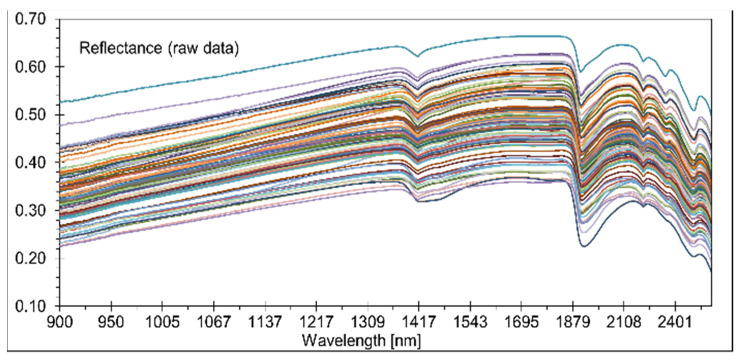
Spectral reflection curves for all soil samples.

The reflection curves are represented by the different soil properties, which differ in their position and composition. The shapes of the curves vary between 0.2 and 0.6. Changes of a darker and more whitish color with distinctly different absorbance characteristics [43] are observable here (Figure 4).

The same figure shows that the spectral reflectance increased gradually with wavelengths from 900–1400 nm. The reflectance reached its first reflection peak between 1390 and 1400 nm. Three more significant reflection peaks appeared at 1450, 1860, and 2120 nm. The two reflection peaks at 1420 and 1930 nm were also characteristic of soil curves. The reflection curves were affected by color, particle size, moisture, and mineral composition (especially the presence of iron). The soil reflectance curves in the NIR region for different soil types, along with the most critical parts for water and carbon and their responsible chemical groups, are shown in Vasques et al. [14,15].

**Figure 5:**
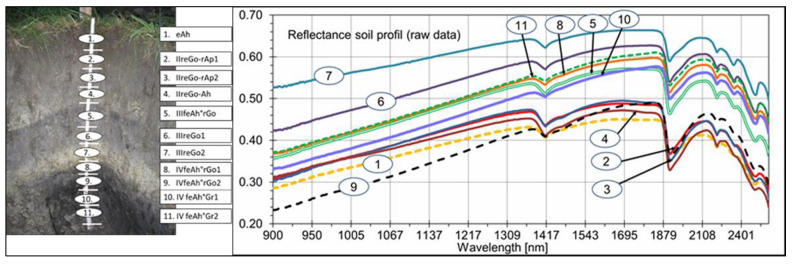
Spectral behavior of the different horizons and vertical discontinuities of the investigated soil profile.

According to Demattê et al. [46], Stenberg et al. [47,48], and Whiting et al. [49], the bands at 1400, 1900, and 2000 nm are produced by OH-groups and water molecule movements, and reflection at 2200 nm indicates the existence of kaolinite and other silicates [50]. Ben–Dor [51] described three major areas for clay minerals in general and smectite minerals, particularly around 1300–1400, 1800–1900, and 2200-2500 nm. According to Dotto et al. [17], the reflections around 1400–1900 and 2200–2500 nm for 1:1 minerals (kaolinite) are relatively strong, while the signal at 1900 nm is very weak. The soils from this investigation were formed from complex alluvial sedimentation interrupted by the building of A-layers. The spectral behavior of the soil horizons and vertical discontinuities varied mainly according to the Ct content, particle size distribution (mainly sand distribution and clay mineral composition), and the condition of Fe oxides (oxides and oxyhydroxides). Fe oxides exist independent of the degree of reduction in these gleyic soils. To clarify this context, in Figure 5, the soil profile is contrasted with the spectral signatures for the 11 soil horizons and vertical discontinuities. The following findings can be derived:

- Variation of the Corg content between the surface and subsurface parts caused a distinct differentiation in spectral shapes. Higher Corg content yielded reduced reflectance intensity along with the spectra, recognizable until horizon 4. A similar relationship was detectable at horizon 9 with an Ah-horizon and Corg content of nearly 1.7%;
- At horizons 10 and 11, higher values of Ccal produced increased spectral readings over the whole reflection shape. Horizons 5 and 8 indicated a more median position;
- The influence of Fe-oxides and -hydroxides was not detectable. Higher Fe content would produce lower reflection intensities. The strong influence of Corg (and also from Ccal, in part) were the main factors that hindered recognition of the interference between the spectrum and the possible occurrence of these minerals [52];
- The same conclusions can be drawn for the case of sand. The effect of quartz particles on spectral behavior was restricted by humus and carbonatic coatings;
- To summarise, Corg and Ccal dominated the spectral shapes. A sandier horizon, which can reflect more energy, was not recognizable. Increasing clay values with increasing depth was also not clearly detectable.

### 3.2. Model Development

Multivariate calibrations were carried out with PLSR and leave-one-out cross-validation procedures for the 55 pre-processed spectra.

#### 3.2.1. Influence of the Pre-Treatment Techniques on the Readings

Pre-processing techniques were used to improve the spectral characteristics and optimize the relationships with the soil properties of interest. As described before, these techniques were divided into the data treatments of smoothing, the degree of the search window, derivation, and additional techniques.

An evaluation of the relevant literature indicates that there is no single or combined pre-processing technique(s) that is optimal for all datasets (Table 1, [14,15,47,48]). Thus, the type and degree of pre-processing methods are data-specific. The same pre-processing was used for all spectra. Soil spectra were first reduced to 900–2659 nm to eliminate severe noise at the edges of each spectrum. The shapes of all pre-treatment procedures are shown in the Supplementary Materials (Figure S11).

Smoothing and degree of the search window: With smoothing, noise in the spectral signals is reduced, and the calibrations become more robust and more straightforward [53,54,55]. The first models were used as a ‘control treatment’, where the raw reflectance values were not smoothed (None-0-raw-) or only weakly smoothed (SG3-SG1- and SG3-SG2-). The next modeling stages included the smoothing technique “moving average”, divided into calculations with search windows of 11 and 25 (MA11-raw- and MA25-raw-). The third modeling stage calculated smoothing according to the Savitzky–Golay method, divided into search windows of 11 and 25 points (SG11-raw- and SG25-raw-). While a “moving average” build involves values within a window of data points, Savitzky–Golay applies a polynomial to fit the data points within a window of points.

The unsmoothed raw data indicated (None-0-raw-, Figure S1, Supplementary Materials) small fluctuations with a maximum of ±0.01 nm, particularly in the lower reflectance ranges (up to about 1100 nm). It is assumed that most of these noise variations in this study are device-related issues caused by light scattering due to sample surface topology, particle size, and possible sample quantity variations in the sample containers. Such effects were described by Martens et al. [53].

Under smoothing transformations with increasing search windows, these variations were distinctly reduced. The number of the readings in the windows determines how many adjacent readings will be used to build an average value. Ranges of 5 to 9 nm have been frequently used in the literature [17,30,31] and a value of 101 nm [36]. Igne et al. [54] used window sizes of 25, as in this study. The smoothing effect was evident in all further transformations based on these smoothings (Supplementary Materials, pre-processing techniques MA11 and MA 25, Figures S4 and S5; SG11 and SG25, Figure S6–S11). Although the reflectance spectra became much less complex, the variation in some of the spectra appeared to be exaggerated, and the others became more similar [53].

Derivation: Within every classification for the smoothing and search windows, the following derivation levels were selected: No derivation, Savitzky–Golay first derivative, first-order polynomial, Savitzky–Golay second derivative, and second-order polynomial.

Spectral derivatives were applied to eliminate the additive and multiplicative effects from the reflectance spectra [27,28]. The most basic derivation method is a slope calculation between two subsequent spectral measurement points; the second-order derivative is calculated by the difference between two successive points of the first-order derivative readings [20]. Calculation of the Savitzky–Golay derivative value at point i within the spectra was carried out using a polynomial. In the Unscrambler software, the selection of the polynomial degree is necessary.

However, Viscarra Rossel and Lark [55] noted that the application of derivations increased the fluctuations by calculating the gradients between adjacent readings. The results yielded strongly fluctuating values (Supplementary Materials, pre-processing techniques Figures S2 and S8). These fluctuations were also increased by the second derivation (Supplementary Materials, pre-processing techniques Figures S3 and S9). Therefore, not all the pre-processing methods were appropriate to improve the accuracy.

Consequently, a smoothing algorithm is often used together with the derivative [55]. This effect is particularly evident here in the first derivation with curve progressions, which indicate fluctuations. Rinnan et al. [20] used a smoothing procedure before calculating the derivatives to reduce the detrimental influence of conventional finite-difference derivatives on the signal-to-noise ratio.

In the current evaluation, the applications using derivations with low smoothing did not deliver the desired weakening of the peak values. The same applies to the second derivatives with prior smoothing, independent of the degree of smoothing. In both cases, the derivations produced a considerable amount of noise.

Additional techniques: Dotto et al. [17] classified multiplicative scatter correction (MSC), detrending, standard normal variate (SNV), and mean centering into the group of scatter-corrective pre-processing methods. In Figure S1, Supplementary Materials, the similarity between SNV and MSC’s curve progression is observable, albeit with different reflection scales. The signal correction procedures are the same for SNV and MSC [56]. SNV is designed to center the underlying linear slope of each sample spectrum.

#### 3.2.2. Selection of the Best Pre-Processing Technique

**Figure 6:**
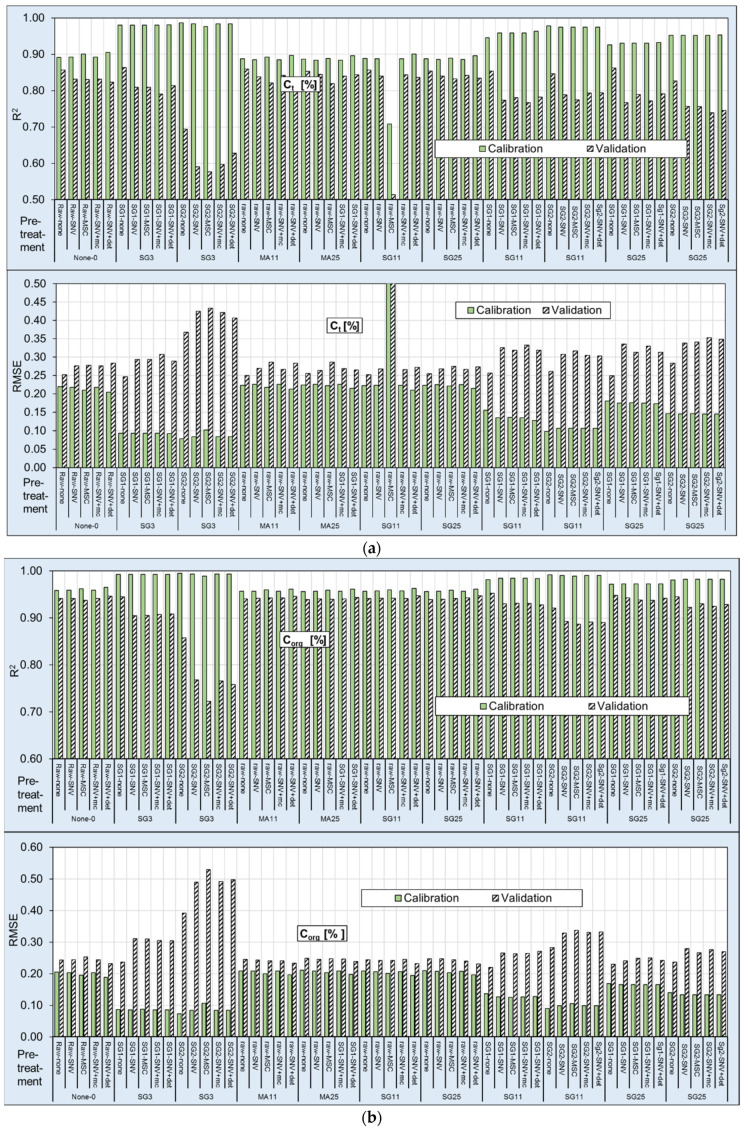

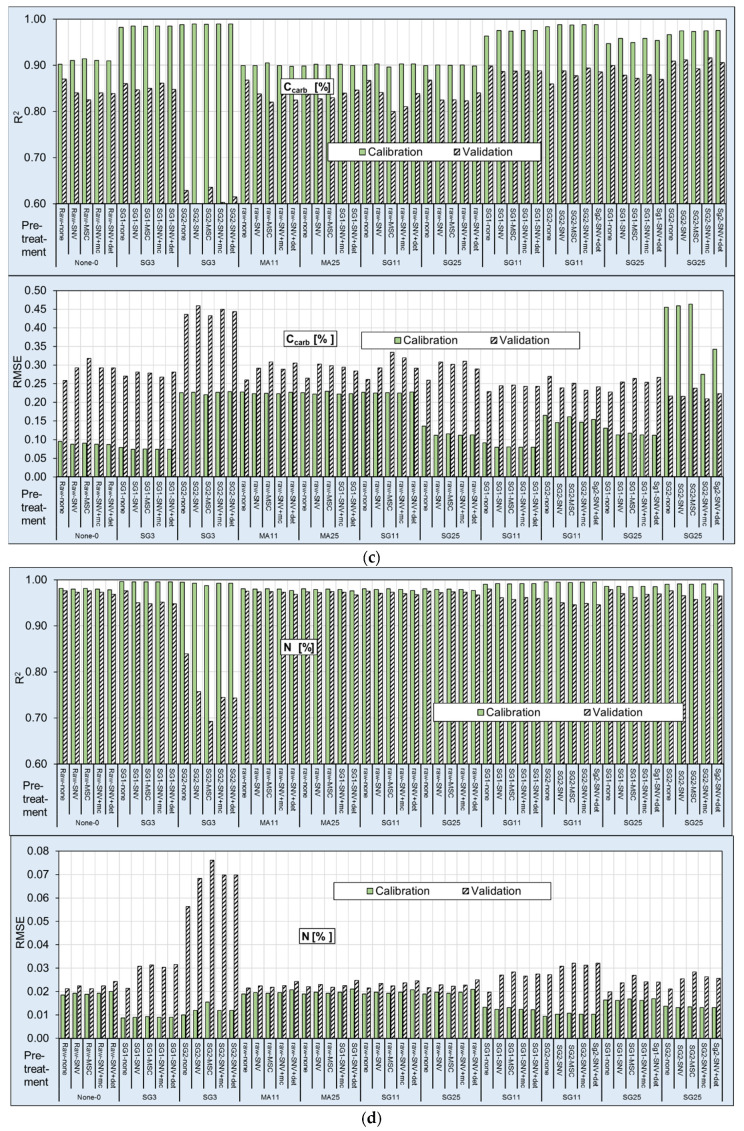

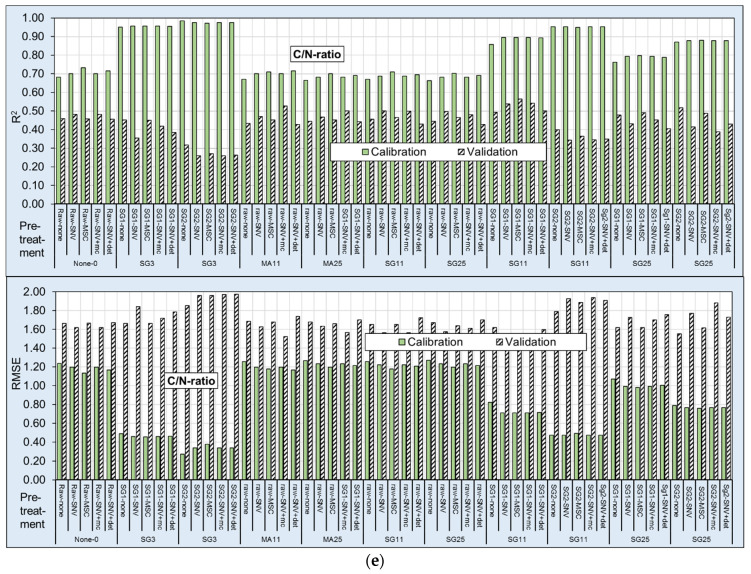
Indices for calculations with partial least squares regression (PLSR) R^2^ and root mean square error (RMSE) for all applied pre-treatments; (**a**) Ct, (**b**) Corg, (**c**) Ccal, (**d**) N, (**e**) C/N. For abbreviations, see Table 3.

The predictive statistics of all soil properties models are shown in Figure 6 and Supplementary Materials, Table S1. The different pre-treatment techniques were evaluated by their potential to predict the single soil parameters. The results showed that the different pre-processing methods had considerable effects on the performance of the selected parameters.

Ct: The pre-treatment trials showed that weak SG-smoothing combined with the Savitzky-Golay first derivative, first-order polynomial, and no additional technique (SG3-SG1-none) provided the best performance for Ct. With this pre-processing transformation, the PLSR models accurately predicted Ct, with R^2^ values of 0.98/0.86 (calibration/validation) and RMSE values of 0.09%/0.25% (calibration/validation). The high RPD and RPIQ values of 7.16/10.92 and 2.58/4.08, respectively, for calibration/validation, were another indication of the model’s quality. The results using the procedure of the Savitzky–Golay second derivative, second-order polynomial, and 11 smoothing segments without an additional technique (SG11-SG2-none) were slightly worse. These results compared well to previous work by other researchers. The studies by Vasques et al. [14,15], Sarkhot et al. [57,58], McDowell et al. [24,25], Knox et al. [30] and Pinheiro et al. [31] described Ct prediction models with lower R^2^ and RPD values (R^2^ 0.86–0.95). A similar R^2^ value (0.97) was also found in work by Reeves et al. [59].

Ccalc: For Ccalc, the calculation SG25-Sg2-SNV+det provided excellent calibration and validation accuracy, with an R^2^ between 0.98 and 0.91 (calibration/validation) and an RMSE of 0.11 and 0.22. Both RPD and RPIQ presented levels with 6.3/7.83 and 3.23/4.11. Good model performance was also obtained using the treatments SG11-SG1, SG11-SG2, SG25-SG1, and SG25-SG2 with the other additional technique.

Corg: Here, the best performance was produced with the pre-treatment of SG3-SG1- none. The R^2^ values of 0.99/0.94 (calibration/validation) and RMSE values of 0.09%/0.24% (calibration/validation) in combination with high RPD and RPIQ values of 11.59/22.41 and 4.19/8.42, respectively, for calibration and validation, indicated high quality. The results of the same procedure with the additional technique were slightly worse. The pre-treatment groups SG3-SG2 and SG11-SG2 also provided good predictions.

The models developed for organic C values in the literature provided slightly worse results [17,21,22,23,26,27,28,32,60]. These differences may have been influenced, at least in part, by the larger range of Corg values in this calculation (compare Table 1 and Figure 3).

Several studies reported that vis–NIR relates better to Corg than NIR alone (Viscarra Rossel et al. [2,3]. Islam et al. [61] noted better results for Australian soils, and Fystro [62] derived a similar result with Norwegian soils. However, in the literature, there are also contrary results. The inclusion of reflection values in the visible area (300–750 nm) delivered only a weak improvement in Swedish soils (Stenberg, 2010 [47,48]. Contrary results were also described by Dunn et al. [63] for soils from SE Australia and the USA. The observation that higher Corg content combines with a darker color is indeed correct, but it seems that this color is only reflected in the spectral signature if the soils come from the same geological substrate. Hummel et al. [64] noted that soil properties such as moisture, texture, and mineralogy could influence the brightness of soils and the same content of Corg.

The combined application of NIR with MIR has also often been discussed. According to Bellon–Maurel and McBratney [65], MIR generally provides slightly better results. The higher sensitivity of NIR than MIR to instrumental errors yielded better predictions with MIR.

N: Of all the parameters, N was the best for calculations. The R^2^ values of the calibration were in no cases worse than 0.9. The best performance was reached with the procedure of SG3-SG1-none, with R^2^ values of 0.99/0.98 (calibration/validation) and RMSE values of 0.01%/0.02% (calibration/validation). The high RPD and RPIQ values of 15.83/30.67 and 6.36/12.39, respectively, for calibration/validation also indicated the model’s quality.

C/N: All results here were insufficient. The best results were calculated with the procedures of SG3-SG1 and SG3-SG2 followed by SG11-SG2. While the R^2^ of the calibration was 0.95 combined with an RMSE of 0.3–0.5 and an RPD/RPIQ of 4.5–8.0 and 2.3–4.2, the results of the validations were weaker. A similar level between calibration and validation was not observed. Therefore, the calculations were not useable.

#### 3.2.3. Description of the Prediction-Relevant Wavelengths

**Figure 7:**
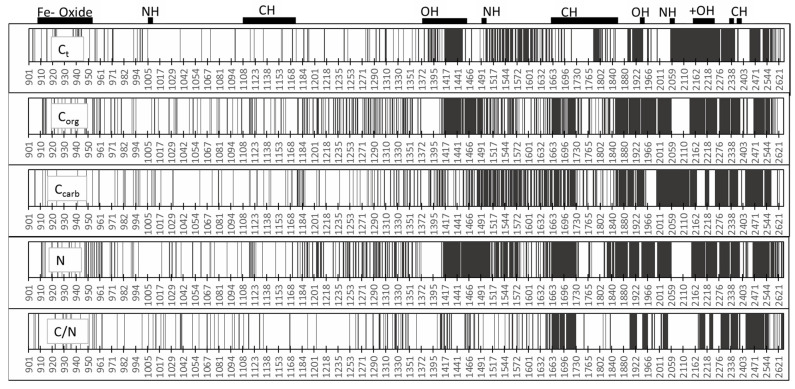
Important wavelengths for the prediction of Ct, Corg, Ccarb, and C/N. Specific reflection wavebands for the different areas for soil are specified on the top (modified from [73]).

Figure 7 illustrates the most important wavelengths for the prediction of C and N. Notably, up to about 1400 nm, single wavelengths were most important. In contrast, above this value, the number of determining wavelengths/wavebands increased, and whole ranges became important. In the case of N and Corg, the regions of 1420–1730, 1840–2060, and 2160–2600 nm were nearly all (without any gaps) relevant for the predictions.

The prediction of Ccarb also indicted relevant wavelengths mainly above 1490 nm (1490–1730, 1850–1970, 2000–2100, 2218, and higher than 2280 nm). The number of selected wavelengths for the C/N ratio was lower than that for the other parameters. The ranges of 1660–1730, 1900–2050, and above 2170 corresponded to the important values of N and Corg.

Several authors have identified the most important single wavelengths and ranges of wavelengths for the prediction of Corg and N. Reflection values around 1100, 1600, 1700–1800, 2000, and 2200–2400 nm have been often described as important for Corg and N calibrations [47,48,64,65,66,67,68,69]; cited the range from 1650 to 2500 as the most relevant for measuring organic C. According to Mouazen et al. [70], the most significant wavelengths for Corg are in the VIS wavelength range (439, 490, and 661 nm) and the NIR region (1109, 1232, 1414, and 1522 nm). Other researchers have found the VIS range (400–700) to be important for the detection of Corg [10,71].

These values from the literature partly differed from those in the present evaluation. However, it was not easy to make specific assignments using the NIR spectrum because other different organic and inorganic molecules could be absorbed in these areas. This was especially true for wavelengths above 2000 nm. Kuang and Mouazen [72] provided other reasons why the important wavelengths can differ. For example, the concentration level and range influence the selected wavelengths. The wavelengths also depend largely on the variability of different soil types in connection with the geological source substrate and type of cultivation [47,48,73].

In this context, Bellon–Maurel and McBratney [65] studied whether discrete variables are sufficient for the relevant calculations. The authors concluded that if the RPD values are over 2 and the R^2^ values are over 0.75, the models are sufficient. An additional option here was the development of low-cost wavelength-based sensors to measure organic C.

## 4. Conclusions

The statistics obtained in this study for calibration, cross-validation, and validation showed that NIRS techniques could effectively provide fast and accurate predictions of Ct, Corg, Ccarb, and N.

The number of publications on the application of NIR spectroscopy in soil science has increased in recent years, as this technique has the advantage of a rapid application without intensive sample preparation. Altogether, 55 procedures of pre-treatments were calculated to determine the best soil sample model from a humus–clay-rich soil. In all calculations, the derivatives with Savitzky–Golay produced the lowest errors and enhanced the pre-treatments, such as detrending; MSC and SNV were not required. Derivatives using Savitzky–Golay calculated by differences were very popular to perform baseline corrections and enhance weak signals. However, in the current study, up to a wavelength of about 1400 nm, this procedure yielded severe fluctuations due to scattering in the original data. Thus, the condition of the measurement device, the sample presentation, the replications, and the accuracy during measurement have an enormous impact on the final results. Only an iterative process can help develop the best models. Smoothing can reduce these fluctuations, but up to a search window of 25, the effect was very low in the case of Ct, Corg, and N, while only Ccarb and C/N indicated an improvement. Important wavelengths for the predictions were mainly in a range of about 1400–2500 nm.

Further work will be undertaken to improve prediction accuracy by applying non-linear calibration techniques such as support vector machine, random forest.

## Figures and Tables

**Table 1:** Overview of applied evaluations with near-infrared reflectance (NIR) spectroscopy, mainly during the last decade, including their target parameters, spectral ranges, pre-processing techniques, and results.

References	Target Parameter	Range Target Parameter	Spectral Range [nm]	Area of Investigation	Soil Sampling Depth	Tested Pre-Processing Technique	RMSEPcal/R^2^ of Best Pre-Processing Technique
				Soil Type			
[14,15]	Ct	0.0072 (0.0017–0.268) %	350–2500	USA, North-central Florida,	180 cm	SG 1st/2nd derivative using a 1st/2nd-order polynomial; normalization; search window 1 to 9	0.17%/0.86 Norris gap derivative with a search window of 7
				Ultisol, Spodosol, Entisol			
[21]	SOC	10.2 (0–55.9) g kg^−1^	350–2500	USA Texas	105 cm	FD	2.8–6.5 g kg^−1^/0.55–0.86 FD
				calcareous, hyperthermic Aridic Ustifluvents			
[22]	SOC	0.9 (0.0–2.7) %	125–2500	Mozambique, Limpopo National Park	2.5–5 cm	Original spectra, original spectra with 1st derivative smoothed 1st derivative, MSC, MSC smoothed, SNV, MSC 1st derivative, MSC smoothed 1st derivative	0.32%/0.83 1st derivative of MSC
				Eutric leptosol, Calcaric cambisol, Arenosols/haplic Luvisol, Ferralic arenosol			
[23]	SOC	1.96 (0.21–6.87) %	350–2500	Brazil, Santa Catarina State	200 cm	CR, NBR, SNV, MSC, ASG, SMO, SG 1st derivative, 1st order polynomial; search window 9	0.48%/0.82 NBR
				Oxisol			
[17]	SOC	1.84 (0.17–4.83) %	350–2500	Brazil, Santa Catarina State	200 cm	Smoothing SG 1st order polynomial; search window 5 CR, DT, BR	0.32%/0.90 CR
	Clay	59.56 (20.9–78.5) %					0.84%/0.62 DT
	Silt	32.94 (16.5–78.0) %					5.26%/0.56 CR
	Sand	7.51(1.0–35.5) %		Oxisol			6.0%/0.33 CR
[24,25]	Ct	10.75 (0.15–55.25) g kg^−1^	400–6000	Main Hawaiin Islands	20 cm	Normalization, SG 1st derivative	2.28%/0.95 normalization, SG 1st derivative
				Andisol, Oxisol, Inceptisol, Ultisol			
[26]	SOC	13.53 (0.79–30.73) g kg^−1^	350–2500	China, Yixing	20 cm	SNV, FD, MSC, WD, SD, MC	2.48 g kg^−1^/0.72 FD, SD
				Different parent materials			
[27,28]	SOC	15.38 (0.79–30.73) g kg^−1^	410–2450	China, Yixing	10 cm	SG smoothing+SG, FD with SG smoothing, SD with SG smoothing, SNV, MC, MSC	2.78/0.73 g kg^−1^ SG
				Zhongxiang Honghu Anthrosol Luvisol Leptosol Gleysol Planosol			
[29]	Cu	5.5–92.2 mg kg^−1^	399–2459	Czech Republic	0–30 cm	SNV, MSC, SG smoothing with a second-order polynomial fit and 11 smoothing points, FD, SD CR	4.0 mg kg^−1^/0.78 FD
	Pb	0.9–55.9 mg kg^−1^		Vertisol, and partly also Chernozem			2.97 mg kg^−1^/0.68 FD
	Mn	41.6–1027.6 mg kg^−1^					97.2 mg kg^−1^/0.6 FD
	Cd	0.0–0.73 mg kg^−1^					0.04 mg kg^−1^/0.80 CR
	Zn	6.6–213.1 mg kg^−1^					13.7 mg kg^−1^/0.77 FD
[30,31]	Ct,	32.0 (1.33–523.3) g kg^−1^	2000–6000 nm	USA, Florida	6 cm	MSC-1st D, SG-Quad, SG-1st D, SG-1st D-Quad, log10(1/x), log10(1/x) SG-1st D	0.23/0.95 log g kg^−1^ SG-Quad
				Spodosol, Entisol, Ultisol, Alfisol, istosol			
	SOC	31.54 (1.33–523.27) g kg^−1^					0.23/095 log g kg^−1^ SG
	RC	21.13 (0.67–502.07) g kg^−1^					0.31/0.93 log g kg^−1^ SG
	HC	0.88 (0.05–19.24) g kg^−1^					0.3/0.86 log g kg^−1^ SG-Quad
[32]	SOC	0.85 (0.01–2.3) %	350–2500	Egypt, Northwestern Sinai peninsula	-	Original spectra, SG smoothing, 1st derivative with SG smoothing, 2nd derivative with SG smoothing, CR, SNV with detrending, MSC, extended MSC	0.19%/0.85 CR
							5.32%/0.90 CR
	Clay	27.22 (0.02–54.3) %					
				Entisol, Aridisol			
[33]	Nt	1.36 (0.21–2.79) g kg^−1^	340–2511	China, Guangdong Province, Conghua District	7 cm	SG smoothing search window 10 with FD, SG smoothing search window 10 with SD, SG smoothing search window 10 with RL	21.61 g kg^−1^/0.82
	Pt	0.75 (0.13–3.15) g kg^−1^					42.84 g kg^−1^/0.79
	Kt	10.55 (0.62–30.39) g kg^−1^		-			25.42 g kg^−1^/0.90; all transformations were used for N, P, and K
[34]	Cd	0.0–1.0 mg kg^−1^	400–2400	China, Sichuan Province	-	MSC with SG smoothing 2nd polynomial search window 7, FD with MSC with SG smoothing 2nd polynomial search window 7, SD MSC with SG smoothing 2nd polynomial search window 7, RL MSC with SG smoothing 2nd polynomial search window 7	1.5 mg kg^−1^/0.77
	Cr	0.0–1000 mg g^−1^					295.7 mg kg^−1^/0.73
	Pb	0.0–1000 mg kg^−1^		Pots texture between sand and loess			67.17 mg kg^−1^/0.71

Ct (total carbon); SOC (organic carbon); RC (recalcitrant carbon; HC (hydrolyzable carbon); OM (organic matter); RMSEPcal/R2 (root mean square error of calibration/coefficient of determination of calibration); SG (Savitzky–Golay); CR (continuum removal); DT (detrend transformation); RL (reciprocal Logarithm); BR (band ratio pre-processing techniques); SNV (standard normal variate); FD (first derivative); MSC (multiplicative signal correction); WD (wavelet detrending); SD (second derivative); MC (mean centering); NBR (normalization by range); SMO (smoothed); MSC-1st D (MSC followed by a five-window smoothing SG filter applied to 1st derivative spectra); SNV (standard normal variate correction, SG = 5 windows smoothing SG filter); SG-Quad (5 windows quadratic smoothing SG filter); SG-1st D (1st derivative-5 window smoothing SG filter); SG-1st D-Quad (1st derivative-5 window quadratic smoothing SG filter); log10(1/x) (absorbance (where x is the reflectance value); log10(1/x) SG-1st D (transformation to absorbance and then application of the 1st derivative—5 window smoothing SG filter); ASG (absorbance and then the application of a Savitzky–Golay derivative).

**Table 2:** Chemical properties and particle size distribution of the soil profile Pulling 8.

	Horizons	Depth [cm]	Ct [%]	N [%]	Ccal [%]	Corg [%]	pH	Clay [%]	Sand [%]	Siltf/m [%]	Siltc [%]	Siltt [%]	Skeleton [%]	KA5	FAO/WRB
1	eAh	0–15	9.04	0.36	5.71	3.37	7.39	62.21	8.39	25.50	3.90	29.41	0.0	Lt3	C
2	II reGo-rAp1	15–20	8.00	0.21	6.03	1.98	7.42	66.75	4.68	26.36	2.22	28.58	0.0	Tt	C
3	II reGo-Ah2	20–30	7.66	0.21	5.69	1.97	7.47	67.28	2.57	27.81	2.33	30.15	0.0	Tt	C
4	II reGo-Ah	30–35	7.37	0.10	5.33	2.04	7.49	69.19	0.93	29.02	0.86	29.87	0.0	Tt	C
5	III feAh°rGo	35–45	8.16	0.12	6.87	1.28	7.51	65.77	0.54	32.62	1.06	33.68	0.0	Tt	C
6	III reGo1	45–54	9.33	0.07	8.65	0.68	7.53	62.08	0.71	34.10	3.12	37.22	0.0	Ts2	C
7	III reGo2	54–62	9.34	0.04	8.82	0.52	7.53	56.64	0.53	35.31	7.52	42.83	0.0	Tu2	C
8	IV feAh°Go1	60–70	9.03	0.08	8.05	0.99	7.53	61.33	0.21	35.19	3.26	38.45	0.0	Tu2	C
9	IV feAh°Go2	70–80	9.29	0.14	7.62	1.67	7.52	61.52	0.41	35.77	2.31	38.08	0.0	Tu2	C
10	IV feAh°Gr1	80–90	9.56	0.14	8.10	1.46	7.52	56.71	0.58	39.73	2.99	42.72	0.0	Tu2	C
11	IV feAh°Gr2	90–100	9.56	0.13	8.27	1.29	7.52	65.40	0.34	32.34	1.90	34.24	0.0	Tt	C

C_t_-Carbon total; C_cal_—C calcareous; Silt_f/m_—Silt (fine+middle); Silt_c_—Silt coarse; Siltt—Sil_t_ total.

**Table 3:** List of all applied combinations of pre-processing techniques for the spectral data.

Smoothing	Search Window	Derivation	Additional Technique	Abbreviation
None	0	Raw data	None	None-0-raw-none
			Standard normal variate	None-0-raw-SNV
			Multiplicative scatter correction	None-0-raw-MSC
			Standard normal variate and mean centering	None-0-raw-SNV+mc
			Standard normal variate and detrending, second-order polynomial	None-0-raw-SNV+det
Smoothing Savitzky–Golay derivative	3	Savitzky–Golay 1st derivative, 1st-order polynomial (within the command “Transform > Derivative > SG” in Unscrambler)	None	SG3-SG1-none
			Standard normal variate	SG3-SG1-SNV
			Multiplicative scatter correction	SG3-SG1-MSC
			Standard normal variate and mean centering	SG3-SG1-SNV+mc
			Standard normal variate and detrending, second-order polynomial	SG3-SG1-SNV+det
Smoothing Savitzky–Golay derivative	3	Savitzky–Golay 2nd derivative, 2nd-order polynomial (within the command “Transform > Derivative > SG” in Unscrambler)	None	SG3-SG2-none
			Standard normal variate	SG3-SG2-SNV
			Multiplicative scatter correction	SG3-SG2-MSC
			Standard normal variate and mean centering	SG3-SG2-SNV+mc
			Standard normal variate and detrending, second-order polynomial	SG3-SG2-SNV+det
Moving average	11	Raw data	None	MA11-raw-none
			Standard normal variate	MA11-raw-SNV
			Multiplicative scatter correction	MA11-raw-MSC
			Standard normal variate and mean centering	MA11-raw-SNV+mc
			Standard normal variate and detrending, second-order polynomial	MA11-raw-SNV+det
Moving average	25	Raw data	None	MA25-raw-none
			Standard normal variate	MA25-raw-SNV
			Multiplicative scatter correction	MA25-raw-MSC
			Standard normal variate and mean centering	MA25-raw-SNV+mc
			Standard normal variate and detrending, second-order polynomial	MA25-raw-SNV+det
Savitzky–Golay, 0-order polynomial (within the command “Transform > Smoothing > SG” in Unscrambler)	11	Raw data	None	SG11-raw-none
			Standard normal variate	SG11-raw-SNV
			Multiplicative scatter correction	SG11-raw-MSC
			Standard normal variate and mean centering	SG11-raw-SNV+mc
			Standard normal variate and detrending, second-order polynomial	SG11-raw-SNV+det
Savitzky–Golay, 0-order polynomial (within the command “Transform > Smoothing > SG” in Unscrambler)	25	Raw data	None	SG25-raw-none
			Standard normal variate	SG25-raw-SNV
			Multiplicative scatter correction	SG25-raw-MSC
			Standard normal variate and mean centering	SG25-raw-SNV+mc
			Standard normal variate and detrending, second-order polynomial	SG25-raw-SNV+det
Savitzky–Golay 1st derivative, 1st-order polynomial (within the command “Transform > Derivative > SG” in Unscrambler)			None	SG11-SG1-none
			Standard normal variate	SG11-SG1-SNV
			Multiplicative scatter correction	SG11-SG1-MSC
	11		Standard normal variate and mean centering	SG11-SG1-SNV+mc
			Standard normal variate and detrending, second-order polynomial	SG11-SG1-SNV+det
Savitzky–Golay 2nd derivative, 2nd-order polynomial (within the command “Transform > Derivative > SG” in Unscrambler)			None	SG11-SG2-none
			Standard normal variate	SG11-SG2-SNV
			Multiplicative scatter correction	SG11-SG2-MSC
	11		Standard normal variate and mean centering	SG11-SG2-SNV+mc
			Standard normal variate and detrending, second-order polynomial	SG11-SG2-SNV+det
Savitzky–Golay, 1st derivative, 1st-order polynomial (within the command “Transform > Derivative > SG” in Unscrambler)			None	SG25-SG1-none
			Standard normal variate	SG25-SG1-SNV
			Multiplicative scatter correction	SG25-SG1-MSC
	25		Standard normal variate and mean centering	SG25-SG1-SNV+mc
			Standard normal variate and detrending, second-order polynomial	SG25-SG1-SNV+det
Savitzky–Golay, 2nd derivative, 2nd-order polynomial (within the command “Transform > Derivative > SG” in Unscrambler)			None	SG25-SG2-none
			Standard normal variate	SG25-SG2-SNV
			Multiplicative scatter correction	SG25-SG2-MSC
	25		Standard normal variate and mean centering	SG25-SG2-SNV+mc
			Standard normal variate and detrending, second-order polynomial	SG25-SG2-SNV+det

SG3, SG11, SG25-Savitzky-Golay, search windows smoothing points 3, 11, and 25; SG1, SG2-Savitzky-Golay, derivative order 1 or 2, polynomial order: 1; SNV-standard normal variate; MSC-multiplicative scatter correction; det-detrending; mc-mean centering.

**Table 4:** Levels of merit for the ratio of the performance deviation (RPD) for the application of NIR spectroscopy [42,44,45].

RPD Value	Classification	Application
0.0–1.9	Very poor	Not recommended
2.0–2.4	Poor	Rough screening
2.5–2.9	Fair	Screening
3.0–3.4	Good	Quality control
3.5–4.0	Very good	Process control
>4.1	Excellent	Any application

## Data Availability

Not applicable.

## Supplementary Materials

The following are available online at https://www.mdpi.com/1424-8220/21/4/1423/s1 Supplementary file “An Evaluation of Different NIR-Spectral Pre-Treatments to Derive the Soil Parameters C and N of a Humus-Clay-Rich Soil_Heil_2021”, Figure S1: Spectra of soils with different pre-processing methods: without smoothing–no derivation (None-0-Raw data); Figure S2: Spectra of soils with different pre-processing methods: smoothing Savitzky–Golay and a search window of 3; Savitzky–Golay 1st derivation and 1st order polynomial (SG3-SG1-); Figure S3: Spectra of soils with different pre-processing methods: smoothing Savitzky–Golay, a search window of 3, Savitzky–Golay 2nd derivation, and 2nd order polynomial; Figure S4: Spectra of soils with different pre-processing methods: a moving average search window of 11 segments and no derivation (MA11-raw-); Figure S5: Spectra of soils with different pre-processing methods: methods: a moving average search window of 25 segments and no derivation (MA25-raw-); Figure S6: Spectra of soils with different pre-processing methods: Savitzky–Golay, 0 order polynomial, a search window of 11 segments, and without derivation (SG11-raw-); Figure S7: Spectra of soils with different pre-processing methods: Savitzky–Golay, 0 order polynomial, a search window of 25 segments, and without derivation (SG25-raw-); Figure S8: Spectra of soils with different pre-processing methods: Savitzky-Golay, 1st derivation, 1st order polynomial, and a search window of 11 segments (SG11-SG1-); Figure S9: Spectra of soils with different pre-processing methods: Savitzky–Golay, 2nd derivation, 2nd order polynomial, and a search window of 11 segments (SG11-SG2-); Figure S10: Spectra of soils with different pre-processing methods: Savitzky–Golay, 1st derivation, 1st order polynomial, and a search window of 25 segments (SG25-SG1-); Figure S11: Spectra of soils with different pre-processing methods: Savitzky–Golay, 2nd derivation, 2nd order polynomial, and a search window of 25 segments (SG25-SG2-); Table S1: Pre-treatments applied to the reflectance spectra with statistics of calibration and validation for different soil properties.
