# Supplementary material for: An Evaluation of Different NIR-Spectral Pre-Treatments to Derive the Soil Parameters C and N of a Humus-Clay-Rich Soil
Source: Sensors (Basel). 2021 Feb 18;21(4):1423. doi: 10.3390/s21041423 (PMC7922103; doi:10.3390/s21041423)
Supplement: Supplementary file 1 [file sensors-21-01423-s001.pdf]

Supplementary material

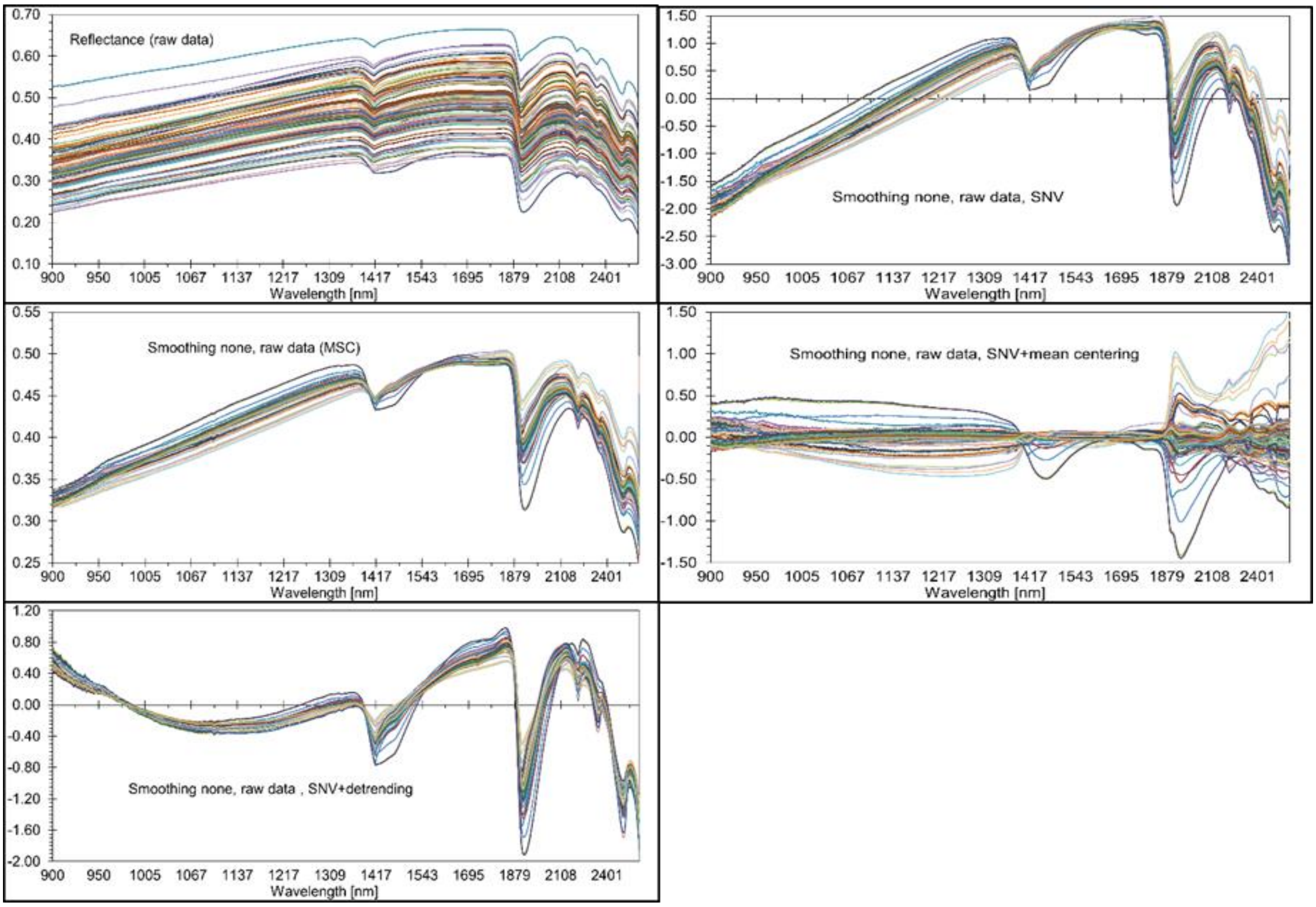

Figure S1. Spectra of soils with different pre-processing methods: without smoothing–no derivation (None-0-Raw data).

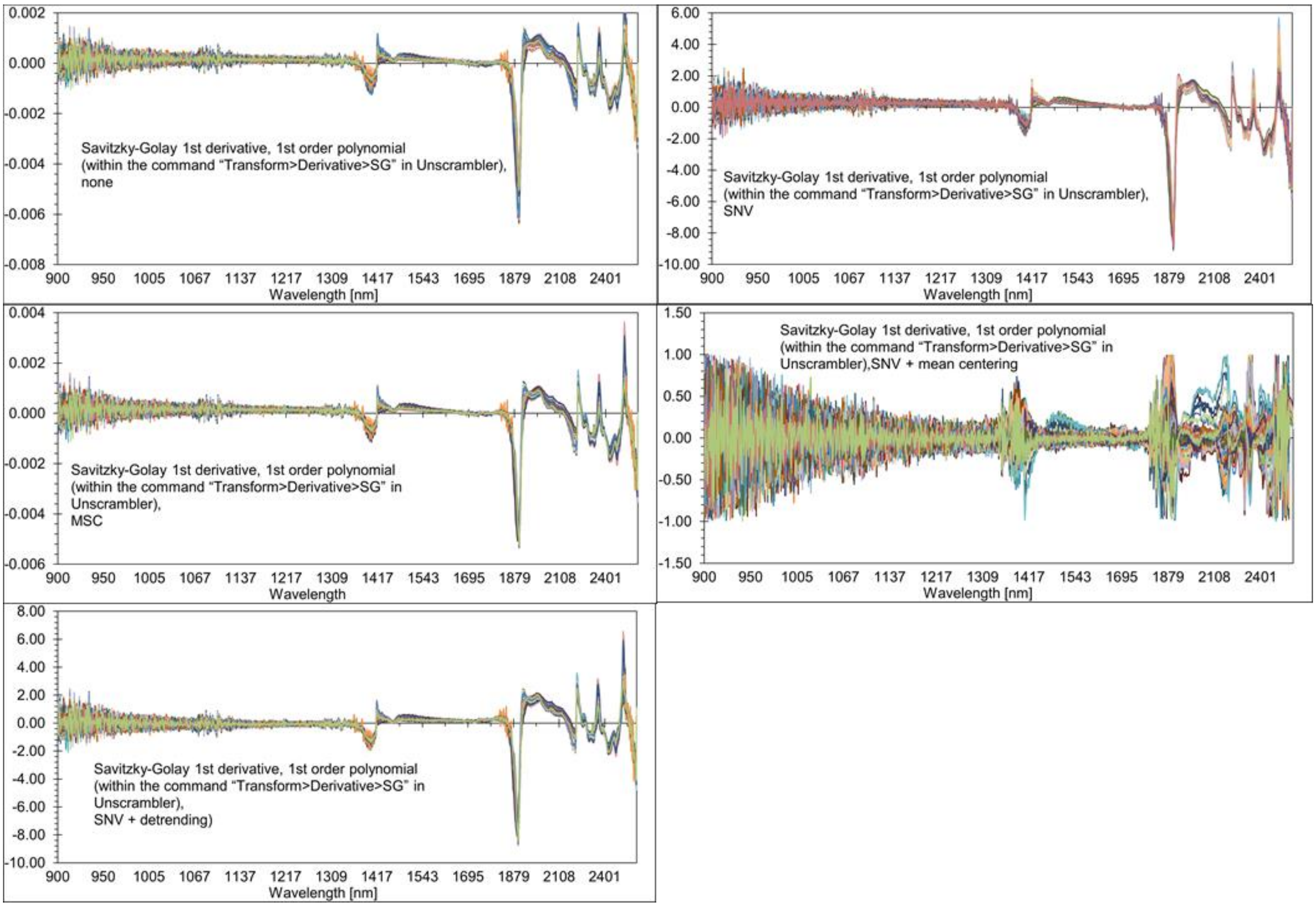

Figure S2. Spectra of soils with different pre-processing methods: smoothing Savitzky–Golay and a search window of 3; Savitzky–Golay 1st derivation and 1st order polynomial (SG3–SG1-).

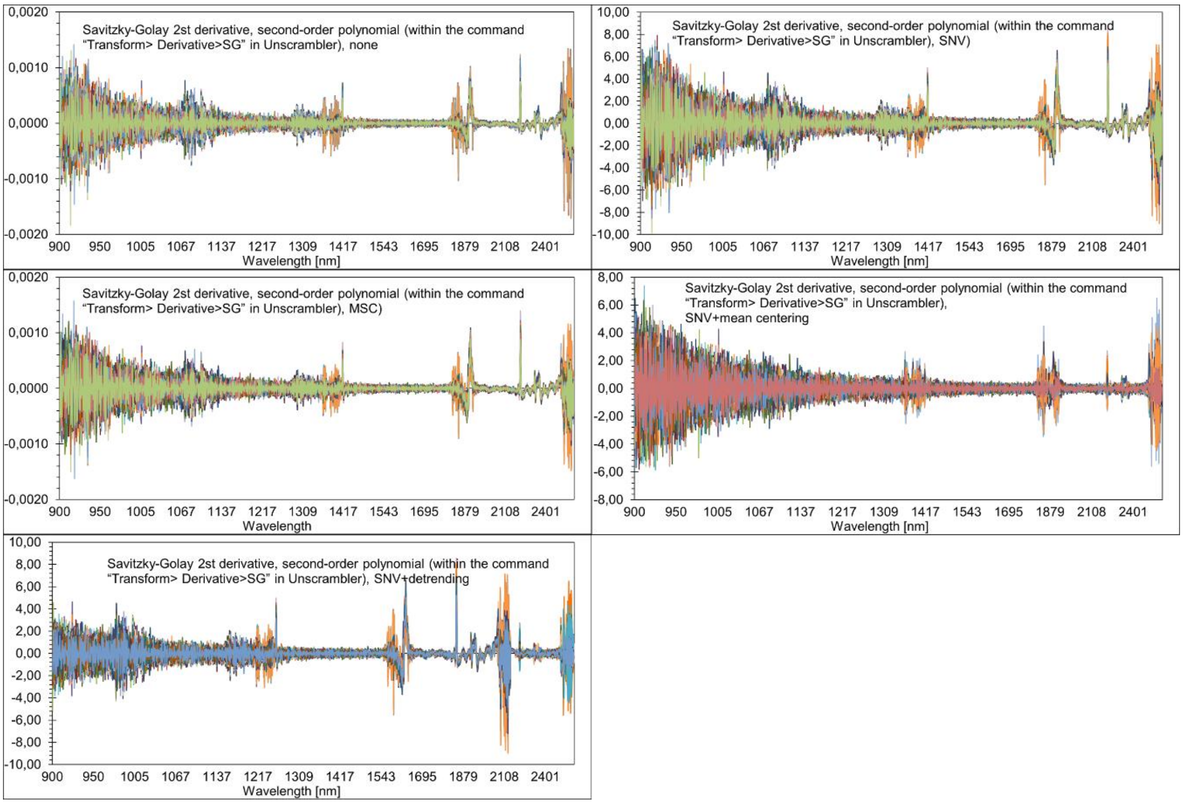

Figure S3. Spectra of soils with different pre-processing methods: smoothing Savitzky–Golay, a search window of 3, Savitzky–Golay 2nd derivation, and 2nd order polynomial.

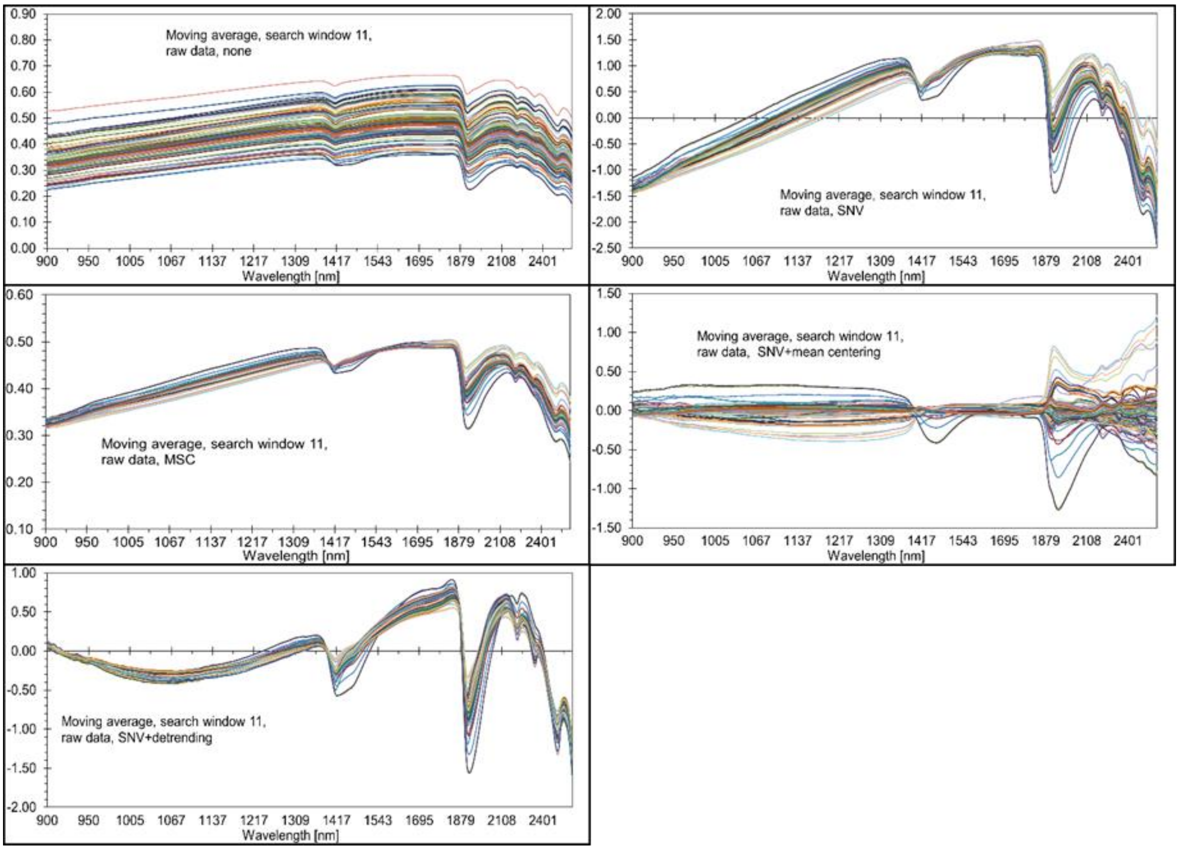

Figure S4. Spectra of soils with different pre-processing methods: a moving average search window of 11 segments and no derivation (MA11-raw-).

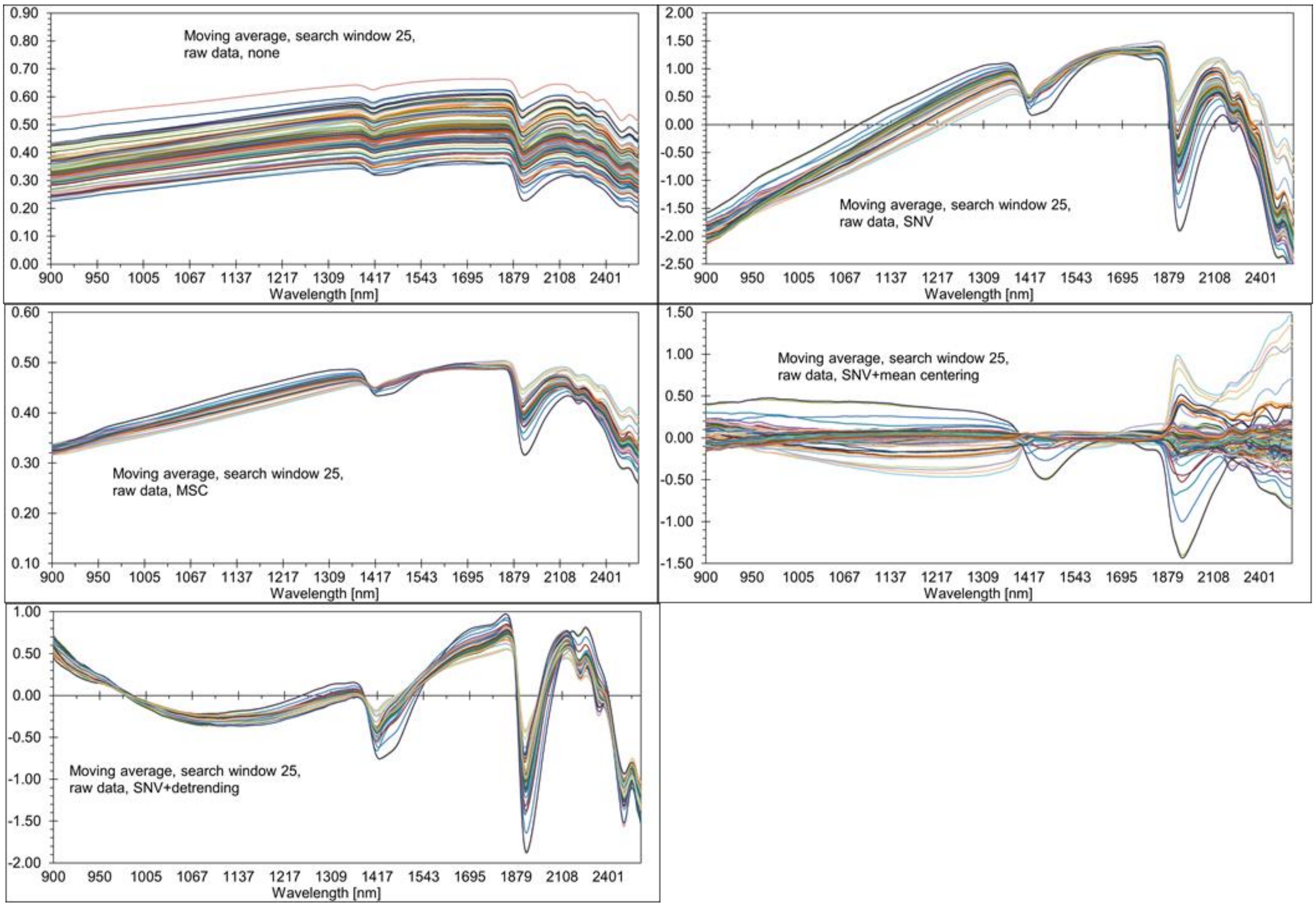

Figure S5. Spectra of soils with different pre-processing methods: methods: a moving average search window of 25 segments and no derivation (MA25-raw-).

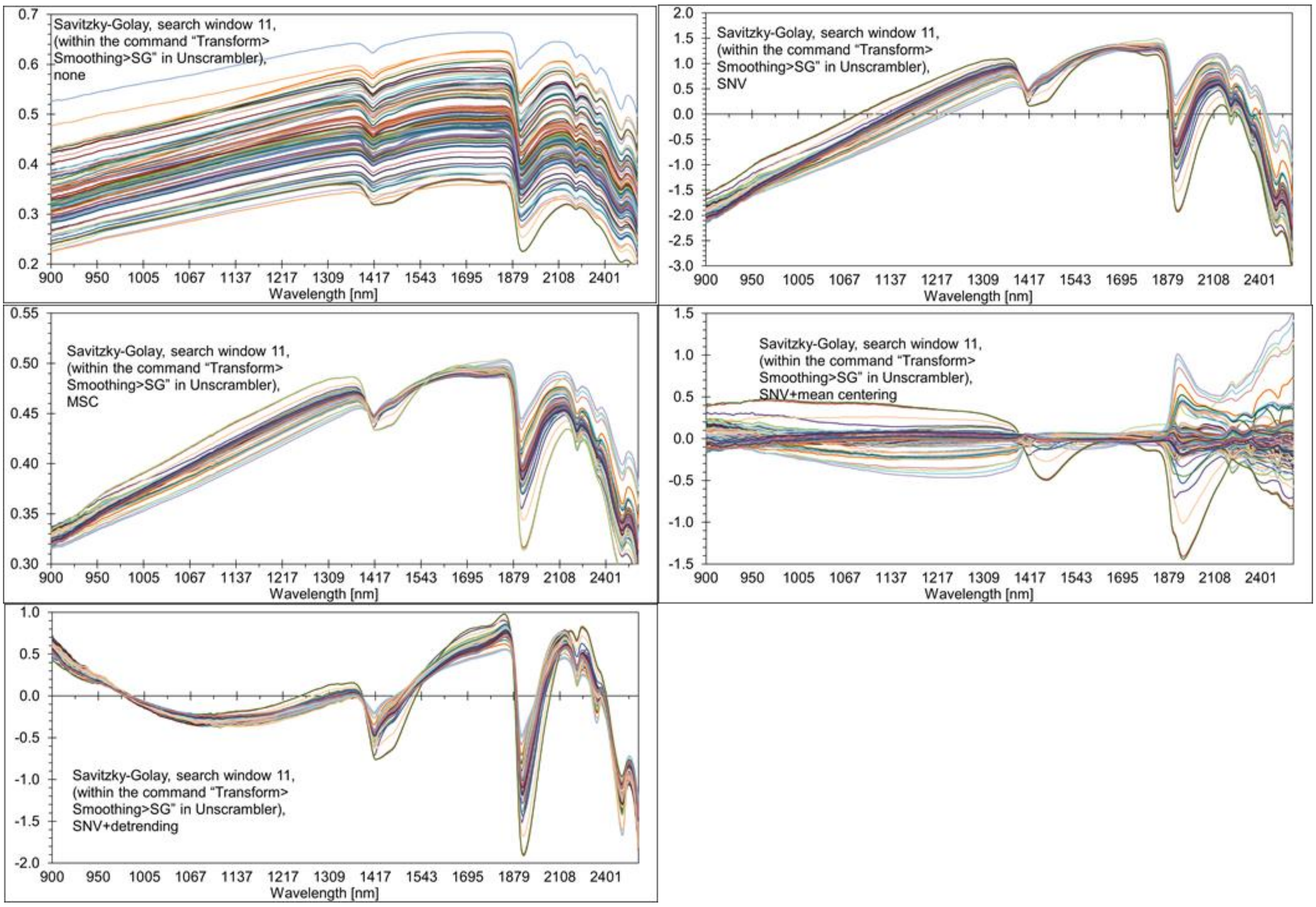

Figure S6. Spectra of soils with different pre-processing methods: Savitzky–Golay, 0 order polynomial, a search window of 11 segments, and without derivation (SG11-raw-).

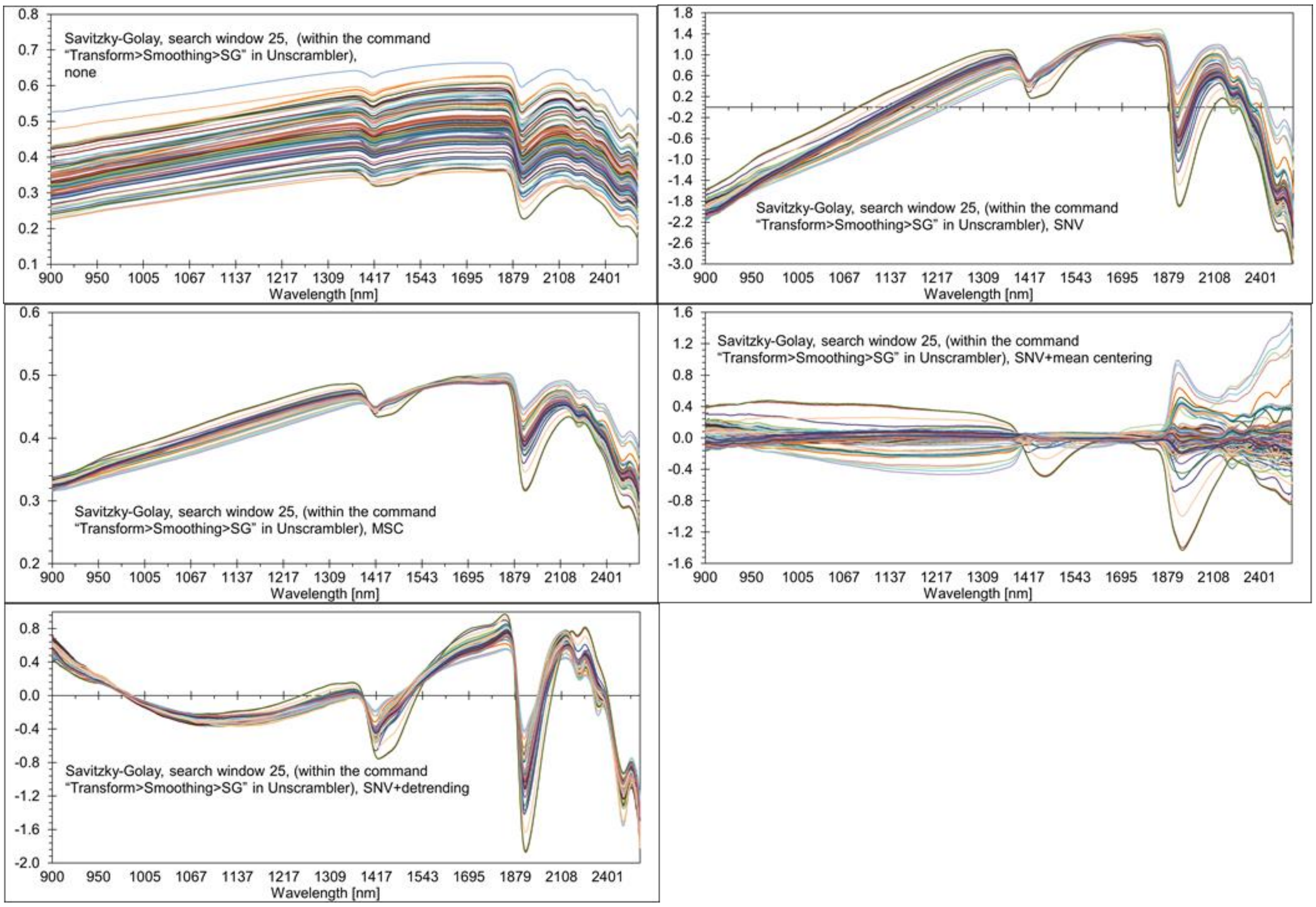

Figure S7. Spectra of soils with different pre-processing methods: Savitzky–Golay, 0 order polynomial, a search window of 25 segments, and without derivation (SG25-raw-).

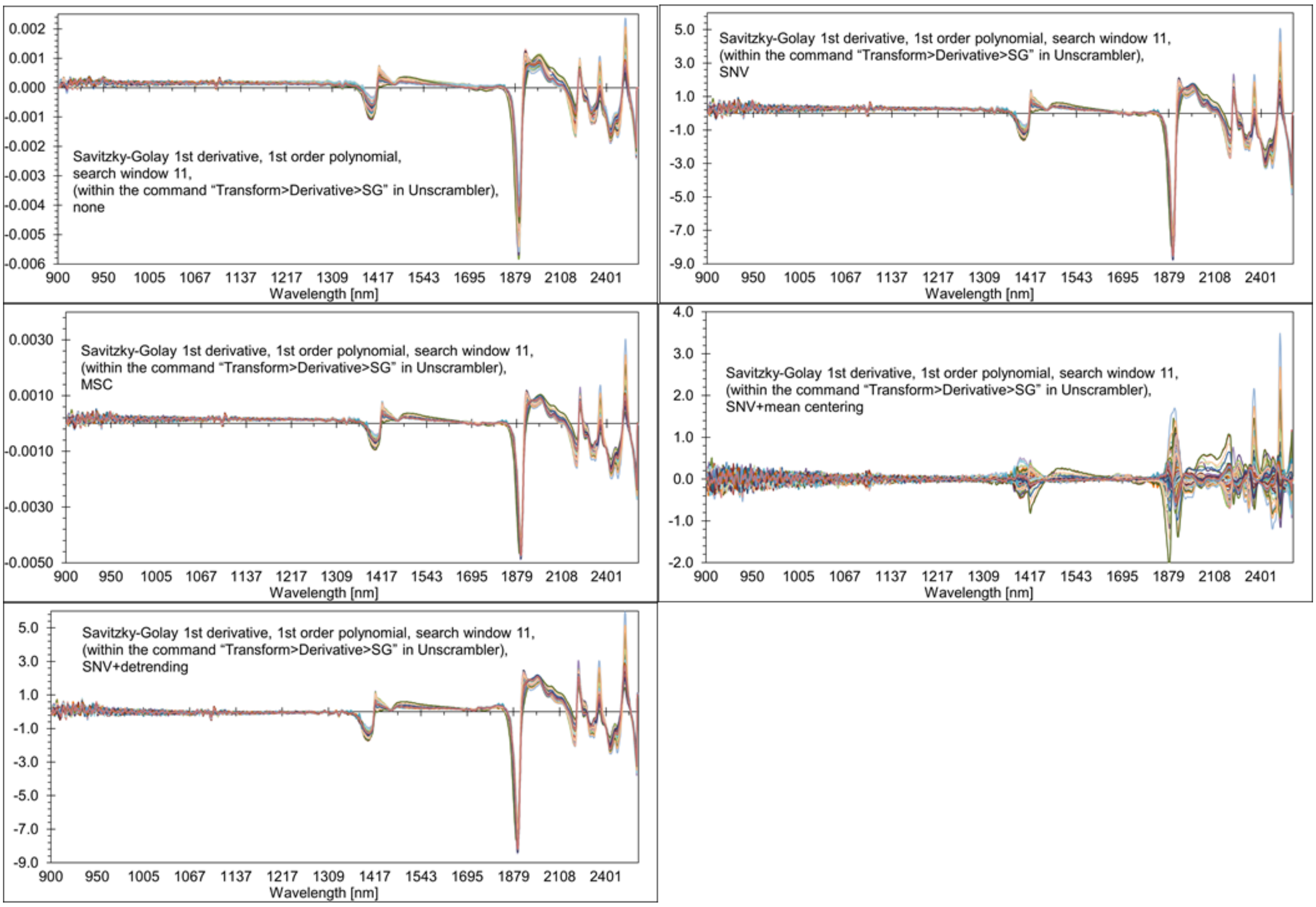

Figure S8. Spectra of soils with different pre-processing methods: Savitzky-Golay, 1st derivation, 1st order polynomial, and a search window of 11 segments (SG11-SG1-).

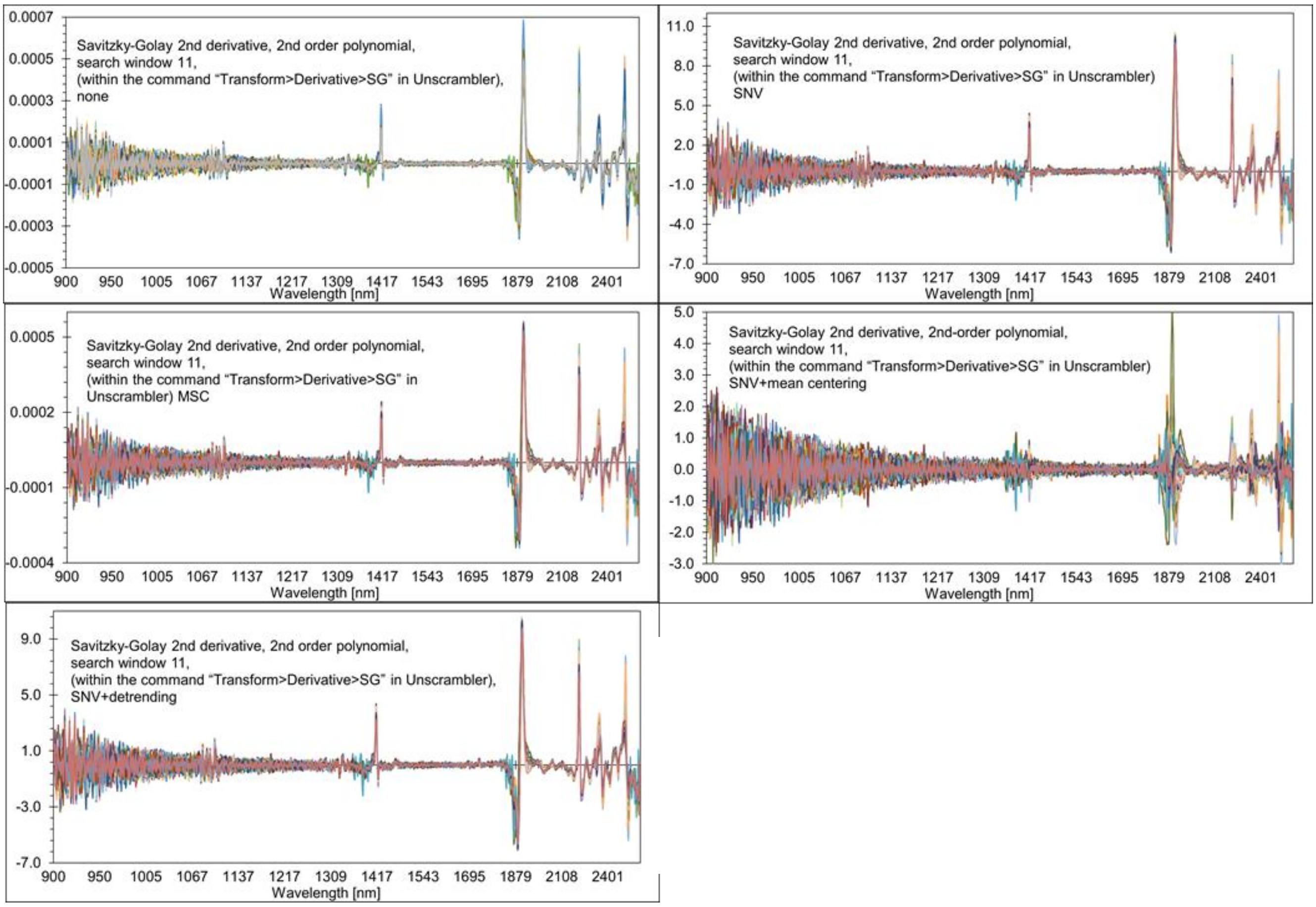

Figure S9. Spectra of soils with different pre-processing methods: Savitzky–Golay, 2nd derivation, 2nd order polynomial, and a search window of 11 segments (SG11-SG2-).

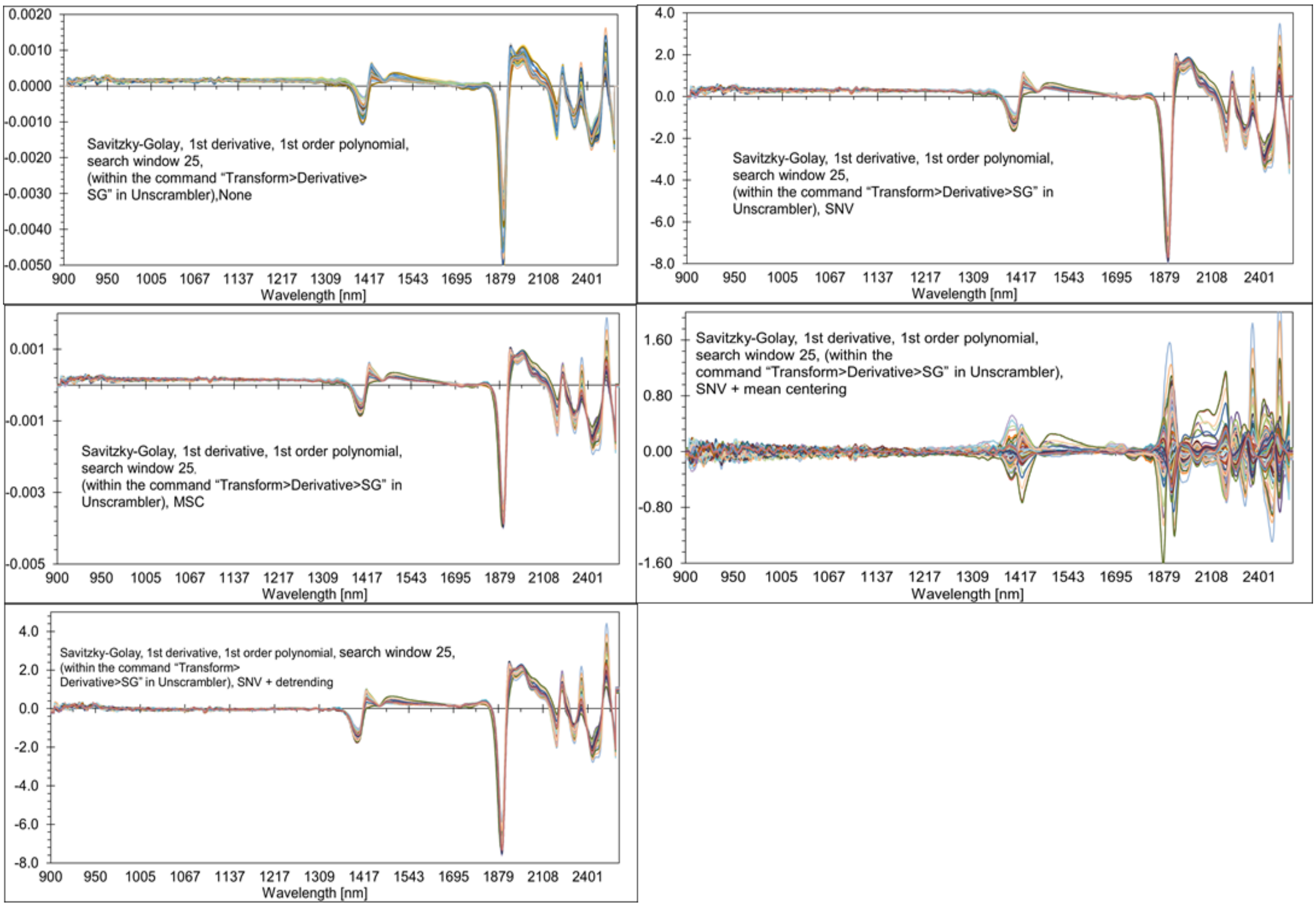

Figure S10. Spectra of soils with different pre-processing methods: Savitzky–Golay, 1st derivation, 1st order polynomial, and a search window of 25 segments (SG25-SG1-) .

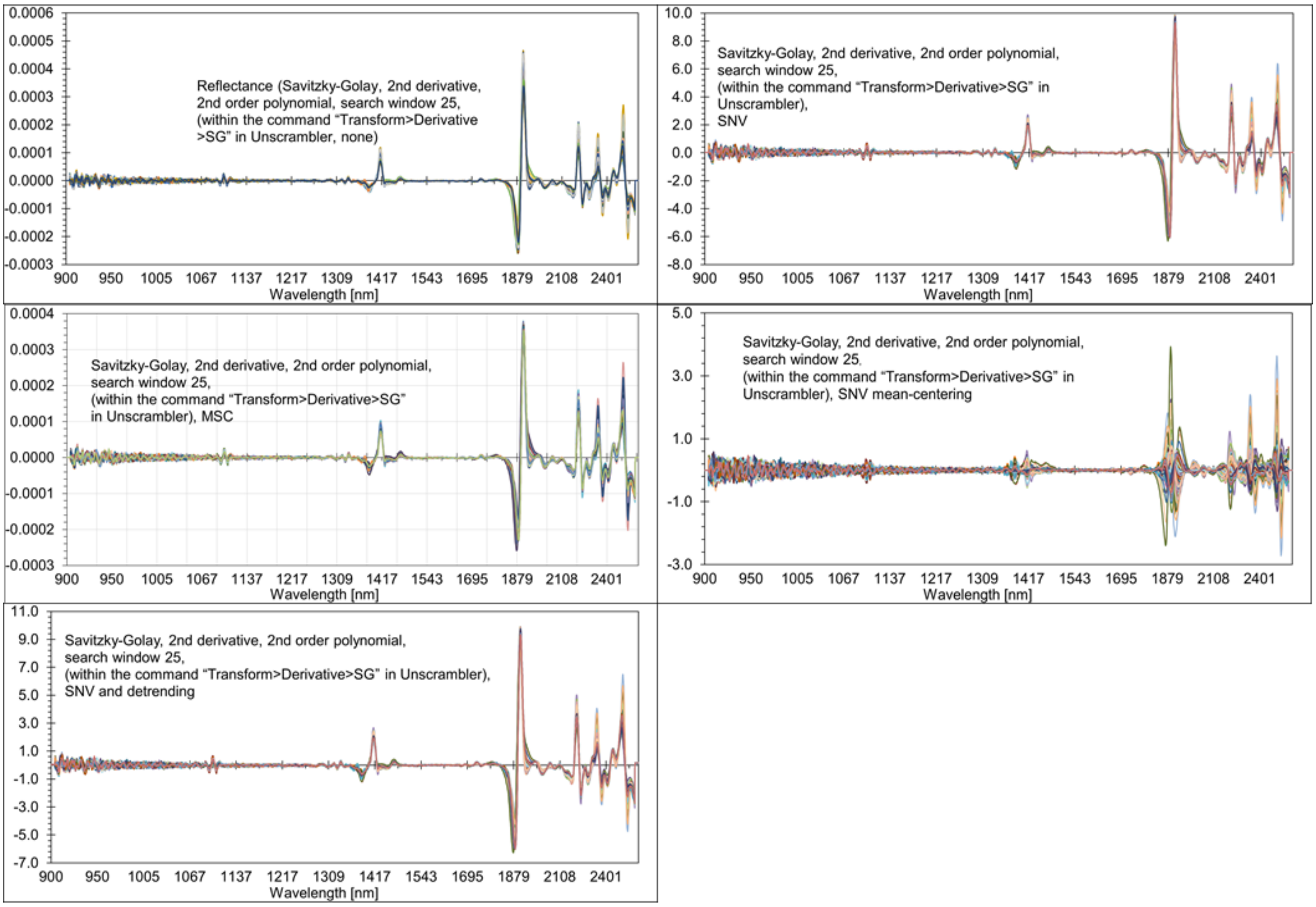

Figure S11. Spectra of soils with different pre-processing methods: Savitzky–Golay, 2nd derivation, 2nd order polynomial, and a search window of 25 segments (SG25-SG2-).

Table S1. Pre-treatments applied to the reflectance spectra with statistics of calibration and validation for different soil properties.

	Smoothing	Search window	Filtering	Enhancing technique	Calibration							Validation						
					R2	RMSE	Bias	SEP	RPD pre	RPD lab	RPIQ lab	R2	RMSE	Bias	SEP	RPD pre	RPD lab	RPIQ lab
Ct	None	0	Raw data	None	0.89	0.22	0.00	0.22	2.87	3.04	4.63	0.86	0.25	0.00	0.25	2.50	2.64	4.03
[%]				Standard normal variate	0.89	0.22	0.00	0.22	2.89	3.06	4.67	0.83	0.28	0.01	0.28	2.35	2.42	3.44
N=120				Multiplicative scatter correction	0.90	0.21	0.00	0.21	3.02	3.18	4.85	0.83	0.28	0.01	0.28	2.35	2.40	3.66
				Standard normal variate and mean centering	0.89	0.22	0.00	0.22	2.89	3.06	4.67	0.83	0.28	0.01	0.28	2.35	2.42	3.44
				Standard normal variate and detrending, second-order polynominal	0.90	0.20	0.00	0.21	3.10	3.26	4.97	0.82	0.28	0.01	0.28	2.30	2.36	3.38
	Smoothing Savitzky-Golay derivative	3	Savitzky-Golay 1st derivative, first-order polynomial (within the command “Transform>Derivative>SG” in Unscrambler)	None	0.98	0.09	0.00	0.09	7.09	7.16	10.92	0.86	0.25	0.00	0.25	2.58	2.71	4.08
				Standard normal variate	0.98	0.09	0.00	0.09	7.09	7.16	10.93	0.81	0.29	0.02	0.29	2.18	2.28	3.37
				Multiplicative scatter correction	0.98	0.09	0.00	0.09	7.07	7.14	10.90	0.81	0.29	0.01	0.29	2.19	2.28	3.35
				Standard normal variate and mean centering	0.98	0.09	0.00	0.09	7.09	7.16	10.93	0.79	0.31	0.02	0.31	2.06	2.17	3.21
				Standard normal variate and detrending, second-order polynominal	0.98	0.09	0.00	0.09	7.17	7.24	11.04	0.81	0.29	0.01	0.29	2.21	2.31	3.38
	Smoothing Savitzky-Golay derivative	3	Savitzky-Golay 2st derivative, first-order polynomial (within the command “Transform> Derivative>SG” in Unscrambler)	None	0.99	0.08	0.00	0.08	8.45	8.51	12.98	0.69	0.37	0.00	0.37	1.44	1.81	3.05
				Standard normal variate	0.98	0.08	0.00	0.08	7.84	7.90	12.06	0.59	0.43	0.00	0.43	1.19	1.57	2.37
				Multiplicative scatter correction	0.98	0.10	0.00	0.10	6.47	6.55	10.00	0.58	0.43	0.02	0.43	1.21	1.54	2.29
				Standard normal variate and mean centering	0.98	0.08	0.00	0.08	7.84	7.90	12.06	0.60	0.42	0.01	0.42	1.22	1.58	2.39
				Standard normal variate and detrending, second-order polynominal	0.98	0.08	0.00	0.08	7.84	7.90	12.06	0.63	0.41	0.02	0.41	1.23	1.64	2.48
	Moving average	11	Raw data	None	0.89	0.22	0.00	0.22	2.82	3.00	4.57	0.86	0.25	0.00	0.25	2.54	2.67	4.05
				Standard normal variate	0.88	0.23	0.00	0.23	2.78	2.96	4.51	0.84	0.27	0.01	0.27	2.39	2.47	3.67
				Multiplicative scatter correction	0.89	0.22	0.00	0.22	2.89	3.06	4.67	0.82	0.29	0.01	0.29	2.30	2.33	3.56
				Standard normal variate and mean centering	0.88	0.23	0.00	0.23	2.78	2.96	4.51	0.84	0.27	0.00	0.27	2.41	2.50	3.71
				Standard normal variate and detrending, second-order polynominal	0.90	0.21	0.00	0.21	2.96	3.13	4.77	0.82	0.28	0.01	0.28	2.31	2.35	3.55
	Moving average	25	Raw data	None	0.89	0.22	0.00	0.22	2.81	2.98	4.55	0.85	0.26	0.00	0.26	2.47	2.61	4.02
				Standard normal variate	0.88	0.23	0.00	0.23	2.77	2.95	4.50	0.84	0.26	0.00	0.26	2.44	2.53	3.82
				Multiplicative scatter correction	0.89	0.22	0.00	0.22	2.83	3.01	4.58	0.82	0.29	0.00	0.29	2.27	2.33	3.60
				Standard normal variate and mean centering	0.88	0.23	0.00	0.23	2.77	2.95	4.50	0.84	0.27	0.00	0.27	2.40	2.49	3.76
				Standard normal variate and detrending, second-order polynominal	0.90	0.21	0.00	0.22	2.94	3.11	4.75	0.84	0.26	0.00	0.27	2.44	2.52	3.84
	Savitzky-Golay, 0-order polynomial (within the command	11	Raw data	None	0.89	0.22	0.00	0.22	2.83	3.00	4.58	0.86	0.25	0.00	0.25	2.51	2.65	4.02
				Standard normal variate	0.89	0.22	0.00	0.22	2.82	2.99	4.57	0.84	0.27	0.00	0.27	2.40	2.49	3.63
				Multiplicative scatter correction	0.71	1.74	0.00	1.75	1.56	1.86	1.83	0.51	2.26	0.13	2.27	1.13	1.43	1.06
				Standard normal variate and mean centering	0.89	0.22	0.00	0.22	2.82	2.99	4.57	0.84	0.27	0.00	0.27	2.46	2.51	3.66
				Standard normal variate and detrending, second-order polynominal	0.90	0.21	0.00	0.21	3.02	3.18	4.85	0.84	0.27	0.01	0.27	2.39	2.45	3.64
	Savitzky-Golay, 0-order polynomial (within the command	25	Raw data	None	0.89	0.22	0.00	0.22	2.82	2.99	4.56	0.85	0.25	0.00	0.26	2.48	2.62	4.04
				Standard normal variate	0.89	0.23	0.00	0.23	2.79	2.96	4.52	0.84	0.27	0.00	0.27	2.40	2.49	3.73
				Multiplicative scatter correction	0.89	0.22	0.00	0.22	2.85	3.02	4.60	0.83	0.27	0.00	0.28	2.35	2.43	3.77
				Standard normal variate and mean centering	0.89	0.23	0.00	0.23	2.79	2.96	4.52	0.84	0.27	0.00	0.27	2.42	2.50	3.75
				Standard normal variate and detrending, second-order polynominal	0.90	0.21	0.00	0.22	2.94	3.11	4.74	0.83	0.27	0.00	0.27	2.36	2.44	3.78
	Savitzky-Golay 1st derivative, first-order polynomial (within the command “Transform>Derivative>SG” in Unscrambler)			None	0.95	0.16	0.00	0.16	4.17	4.29	6.55	0.85	0.26	0.00	0.26	2.54	2.60	3.96
				Standard normal variate	0.96	0.13	0.00	0.14	4.85	4.95	7.55	0.77	0.33	0.02	0.33	2.04	2.05	2.86
				Multiplicative scatter correction	0.96	0.14	0.00	0.14	4.81	4.91	7.49	0.78	0.32	0.01	0.32	2.06	2.09	2.93
		11		Standard normal variate and mean centering	0.96	0.13	0.00	0.14	4.85	4.95	7.55	0.77	0.33	0.02	0.33	2.02	2.01	2.81
				Standard normal variate and detrending, second-order polynominal	0.96	0.13	0.00	0.13	5.13	5.22	7.97	0.78	0.32	0.02	0.32	2.07	2.10	2.83
	Savitzky-Golay 2st derivative, second-order polynomial (within the command “Transform>Derivative>SG” in Unscrambler)			None	0.98	0.10	0.00	0.10	6.71	6.79	10.36	0.85	0.26	0.00	0.26	2.44	2.55	3.80
				Standard normal variate	0.97	0.11	0.00	0.11	6.18	6.26	9.55	0.79	0.31	0.02	0.31	2.03	2.17	3.22
				Multiplicative scatter correction	0.97	0.11	0.00	0.11	6.21	6.29	9.59	0.77	0.32	0.01	0.32	1.94	2.11	3.04
		11		Standard normal variate and mean centering	0.97	0.11	0.00	0.11	6.18	6.26	9.55	0.79	0.30	0.01	0.31	2.07	2.19	3.26
				Standard normal variate and detrending, second-order polynominal	0.97	0.11	0.00	0.11	6.18	6.26	9.55	0.79	0.30	0.00	0.30	2.06	2.20	3.27
	Savitzky-Golay, 1st derivative, first-order polynomial (within the command “Transform>Derivative>SG” in Unscrambler)			None	0.93	0.18	0.00	0.18	3.55	3.69	5.63	0.86	0.25	0.01	0.25	2.63	2.68	4.41
				Standard normal variate	0.93	0.17	0.00	0.18	3.68	3.82	5.83	0.77	0.33	0.02	0.34	2.02	1.99	2.92
				Multiplicative scatter correction	0.93	0.18	0.00	0.18	3.67	3.80	5.80	0.79	0.31	0.02	0.31	2.12	2.13	3.12
		25		Standard normal variate and mean centering	0.93	0.17	0.00	0.18	3.68	3.82	5.83	0.77	0.33	0.01	0.33	2.04	2.03	2.96
				Standard normal variate and detrending, second-order polynominal	0.93	0.17	0.00	0.17	3.73	3.86	5.89	0.79	0.31	0.02	0.31	2.13	2.13	3.21
	Savitzky-Golay, 2st derivative, second-order polynomial (within the command “Transform>Derivative>SG” in Unscrambler)			None	0.95	0.15	0.00	0.15	4.47	4.58	6.99	0.83	0.28	0.00	0.28	2.36	2.35	3.59
				Standard normal variate	0.95	0.15	0.00	0.15	4.48	4.59	7.01	0.76	0.34	0.01	0.34	1.97	1.97	2.90
				Multiplicative scatter correction	0.95	0.15	0.00	0.15	4.46	4.57	6.97	0.76	0.34	0.01	0.34	1.97	1.96	2.91
		25		Standard normal variate and mean centering	0.95	0.15	0.00	0.15	4.48	4.59	7.01	0.74	0.35	0.01	0.35	1.90	1.89	2.78
				Standard normal variate and detrending, second-order polynominal	0.95	0.14	0.00	0.15	4.50	4.61	7.03	0.75	0.35	0.01	0.35	1.93	1.92	2.81

	Smoothing	Search window	Filtering	Enhancing technique	Calibration							Validation						
					R2	RMSE	Bias	SEP	RPD pre	RPD lab	$RPIQ_{lab}$	R2	RMSE	Bias	SEP	RPD pre	RPD lab	$RPIQ_{lab}$
N	None	0	Raw data	None	0.98	0.02	0.00	0.02	7.33	7.40	14.31	0.98	0.02	0.00	0.02	6.39	6.47	12.51
[%]				Standard normal variate	0.98	0.02	0.00	0.02	7.04	7.11	13.75	0.97	0.02	0.00	0.02	6.11	6.13	11.06
N=120				Multiplicative scatter correction	0.98	0.02	0.00	0.02	7.25	7.32	14.16	0.98	0.02	0.00	0.02	6.49	6.50	11.92
				Standard normal variate and mean centering	0.98	0.02	0.00	0.02	7.04	7.11	13.75	0.97	0.02	0.00	0.02	6.11	6.13	11.06
				Standard normal variate and detrending, second-order polynominal	0.98	0.02	0.00	0.02	6.78	6.86	13.26	0.97	0.02	0.00	0.02	5.64	5.65	10.39
	Smoothing Savitzky-Golay derivative	3	Savitzky-Golay 1st derivative, first-order polynomial (within the command “Transform>Derivative>SG” in Unscrambler)	None	1.00	0.01	0.00	0.01	15.83	15.86	30.67	0.98	0.02	0.00	0.02	6.36	6.46	12.39
				Standard normal variate	1.00	0.01	0.00	0.01	15.30	15.34	29.66	0.95	0.03	0.00	0.03	4.37	4.48	8.60
				Multiplicative scatter correction	1.00	0.01	0.00	0.01	14.83	14.86	28.73	0.95	0.03	0.00	0.03	4.29	4.39	8.37
				Standard normal variate and mean centering	1.00	0.01	0.00	0.01	15.30	15.34	29.66	0.95	0.03	0.00	0.03	4.43	4.54	8.72
				Standard normal variate and detrending, second-order polynominal	1.00	0.01	0.00	0.01	15.18	15.22	29.42	0.95	0.03	0.00	0.03	4.29	4.37	8.42
	Smoothing Savitzky-Golay derivative	3	Savitzky-Golay 2st derivative, first-order polynomial (within the command “Transform>Derivative>SG” in Unscrambler)	None	0.99	0.01	0.00	0.01	13.76	13.79	26.67	0.84	0.06	0.00	0.06	2.01	2.44	4.67
				Standard normal variate	0.99	0.01	0.00	0.01	11.49	11.54	22.31	0.76	0.07	0.00	0.07	1.59	2.01	3.68
				Multiplicative scatter correction	0.99	0.02	0.00	0.02	8.85	8.91	17.22	0.69	0.08	0.00	0.08	1.52	1.81	3.29
				Standard normal variate and mean centering	0.99	0.01	0.00	0.01	11.49	11.54	22.31	0.74	0.07	0.00	0.07	1.57	1.97	3.60
				Standard normal variate and detrending, second-order polynominal	0.99	0.01	0.00	0.01	11.50	11.54	22.31	0.74	0.07	0.00	0.07	1.59	1.97	3.60
	Moving average	11	Raw data	None	0.98	0.02	0.00	0.02	7.20	7.27	14.05	0.98	0.02	0.00	0.02	6.34	6.41	12.03
				Standard normal variate	0.98	0.02	0.00	0.02	6.95	7.02	13.58	0.97	0.02	0.00	0.02	6.14	6.15	11.08
				Multiplicative scatter correction	0.98	0.02	0.00	0.02	7.08	7.15	13.83	0.97	0.02	0.00	0.02	6.27	6.31	11.55
				Standard normal variate and mean centering	0.98	0.02	0.00	0.02	6.95	7.02	13.58	0.97	0.02	0.00	0.02	6.10	6.11	11.02
				Standard normal variate and detrending, second-order polynominal	0.98	0.02	0.00	0.02	6.54	6.61	12.78	0.97	0.02	0.00	0.02	5.61	5.65	10.25
	Moving average	25	Raw data	None	0.98	0.02	0.00	0.02	7.15	7.22	13.95	0.97	0.02	0.00	0.02	6.14	6.21	11.63
				Standard normal variate	0.98	0.02	0.00	0.02	6.88	6.95	13.44	0.97	0.02	0.00	0.02	5.99	5.99	10.83
				Multiplicative scatter correction	0.98	0.02	0.00	0.02	7.04	7.11	13.75	0.97	0.02	0.00	0.02	6.25	6.29	11.48
				Standard normal variate and mean centering	0.98	0.02	0.00	0.02	6.88	6.95	13.44	0.97	0.02	0.00	0.02	6.09	6.12	11.06
				Standard normal variate and detrending, second-order polynominal	0.98	0.02	0.00	0.02	6.45	6.52	12.62	0.97	0.02	0.00	0.02	5.51	5.55	10.12
	Savitzky-Golay, 0-order polynomial (within the command	11	Raw data	None	0.98	0.02	0.00	0.02	7.20	7.27	14.05	0.98	0.02	0.00	0.02	6.34	6.41	12.02
				Standard normal variate	0.98	0.02	0.00	0.02	6.88	6.95	13.44	0.97	0.02	0.00	0.02	5.81	5.86	10.60
				Multiplicative scatter correction	0.98	0.02	0.00	0.02	7.08	7.15	13.83	0.97	0.02	0.00	0.02	6.13	6.14	11.24
				Standard normal variate and mean centering	0.98	0.02	0.00	0.02	6.91	6.99	13.51	0.97	0.02	0.00	0.02	5.77	5.81	10.52
				Standard normal variate and detrending, second-order polynominal	0.98	0.02	0.00	0.02	6.55	6.63	12.82	0.97	0.02	0.00	0.02	5.59	5.61	10.30
	Savitzky-Golay, 0-order polynomial (within the command	25	Raw data	None	0.98	0.02	0.00	0.02	7.16	7.23	13.97	0.98	0.02	0.00	0.02	6.32	6.36	11.92
				Standard normal variate	0.98	0.02	0.00	0.02	6.88	6.95	13.44	0.97	0.02	0.00	0.02	6.04	6.04	10.91
				Multiplicative scatter correction	0.98	0.02	0.00	0.02	7.04	7.11	13.75	0.97	0.02	0.00	0.02	6.18	6.20	11.30
				Standard normal variate and mean centering	0.98	0.02	0.00	0.02	6.88	6.95	13.44	0.97	0.02	0.00	0.02	6.06	6.07	10.97
				Standard normal variate and detrending, second-order polynominal	0.98	0.02	0.00	0.02	6.48	6.56	12.69	0.97	0.03	0.00	0.03	5.48	5.49	10.01
	Savitzky-Golay 1st derivative, first-order polynomial (within the command “Transform>Derivative>SG” in Unscrambler)			None	0.99	0.01	0.00	0.01	10.29	10.34	20.00	0.98	0.02	0.00	0.02	6.96	7.00	13.51
				Standard normal variate	0.99	0.01	0.00	0.01	11.08	11.12	21.51	0.96	0.03	0.00	0.03	5.04	5.08	9.69
				Multiplicative scatter correction	0.99	0.01	0.00	0.01	10.47	10.52	20.34	0.96	0.03	0.00	0.03	4.81	4.85	9.18
		11		Standard normal variate and mean centering	0.99	0.01	0.00	0.01	11.08	11.12	21.51	0.96	0.03	0.00	0.03	5.08	5.16	9.85
				Standard normal variate and detrending, second-order polynominal	0.99	0.01	0.00	0.01	11.13	11.18	21.62	0.96	0.03	0.00	0.03	4.94	5.00	9.64
	Savitzky-Golay 2st derivative, second-order polynomial (within the command “Transform>Derivative>SG” in Unscrambler)			None	1.00	0.01	0.00	0.01	14.47	14.51	28.05	0.96	0.03	0.00	0.03	4.97	5.05	9.88
				Standard normal variate	0.99	0.01	0.00	0.01	13.34	13.38	25.87	0.95	0.03	0.00	0.03	4.33	4.47	8.72
				Multiplicative scatter correction	0.99	0.01	0.00	0.01	12.66	12.70	24.56	0.95	0.03	0.00	0.03	4.15	4.29	8.31
		11		Standard normal variate and mean centering	0.99	0.01	0.00	0.01	13.34	13.38	25.87	0.95	0.03	0.00	0.03	4.27	4.41	8.60
				Standard normal variate and detrending, second-order polynominal	0.99	0.01	0.00	0.01	13.33	13.37	25.86	0.95	0.03	0.00	0.03	4.14	4.29	8.36
	Savitzky-Golay, 1st derivative, first-order polynomial (within the			None	0.99	0.02	0.00	0.02	8.32	8.38	16.21	0.98	0.02	0.00	0.02	6.86	6.90	13.36
				Standard normal variate	0.99	0.02	0.00	0.02	8.39	8.45	16.35	0.97	0.02	0.00	0.02	5.73	5.79	11.01
				Multiplicative scatter correction	0.98	0.02	0.00	0.02	8.12	8.18	15.81	0.96	0.03	0.00	0.03	5.10	5.11	9.52
		25		Standard normal variate and mean centering	0.99	0.02	0.00	0.02	8.39	8.45	16.35	0.97	0.02	0.00	0.02	5.61	5.68	10.81
				Standard normal variate and detrending, second-order polynominal	0.98	0.02	0.00	0.02	8.06	8.12	15.71	0.97	0.02	0.00	0.02	5.67	5.73	10.89
	Savitzky-Golay, 2st derivative, second-order polynomial (within the			None	0.99	0.01	0.00	0.01	10.01	10.06	19.45	0.98	0.02	0.00	0.02	6.48	6.52	12.51
				Standard normal variate	0.99	0.01	0.00	0.01	10.41	10.46	20.22	0.97	0.03	0.00	0.03	5.30	5.39	10.38
				Multiplicative scatter correction	0.99	0.01	0.00	0.01	10.21	10.26	19.83	0.96	0.03	0.00	0.03	4.81	4.83	9.31
		25		Standard normal variate and mean centering	0.99	0.01	0.00	0.01	10.41	10.46	20.22	0.96	0.03	0.00	0.03	5.12	5.21	10.04
				Standard normal variate and detrending, second-order polynominal	0.99	0.01	0.00	0.01	10.43	10.48	20.27	0.97	0.03	0.00	0.03	5.31	5.37	10.36

	Smoothing	Search window	Filtering	Enhancing technique	Calibration							Validation						
					R2	RMSE	Bias	SEP	RPD pre	RPD lab	$RPIQ_{lab}$	R2	RMSE	Bias	SEP	RPD pre	RPD lab	$RPIQ_{lab}$
Corg	None	0	Raw data	None	0.96	0.21	0.00	0.21	4.79	4.89	9.42	0.94	0.24	0.00	0.24	4.04	4.14	7.97
[%]				Standard normal variate	0.96	0.20	0.00	0.20	4.85	4.95	9.53	0.94	0.24	0.00	0.24	4.11	4.13	7.39
N=120				Multiplicative scatter correction	0.96	0.19	0.00	0.20	5.06	5.16	9.93	0.94	0.25	0.00	0.25	3.96	3.98	7.31
				Standard normal variate and mean centering	0.96	0.20	0.00	0.20	4.85	4.95	9.53	0.94	0.24	0.00	0.24	4.11	4.13	7.39
				Standard normal variate and detrending, second-order polynominal	0.96	0.19	0.00	0.19	5.25	5.34	10.28	0.95	0.23	0.00	0.23	4.28	4.33	8.01
	Smoothing Savitzky-Golay derivative	3	Savitzky-Golay 1st derivative, first-order polynomial (within the command "Transform>Derivative>SG" in	None	0.99	0.09	0.00	0.09	11.59	11.64	22.41	0.94	0.24	-0.01	0.24	4.19	4.26	8.42
				Standard normal variate	0.99	0.09	0.00	0.09	11.72	11.76	22.64	0.90	0.31	0.01	0.31	3.15	3.23	6.32
				Multiplicative scatter correction	0.99	0.09	0.00	0.09	11.38	11.42	21.99	0.91	0.31	0.01	0.31	3.18	3.25	6.29
				Standard normal variate and mean centering	0.99	0.09	0.00	0.09	11.72	11.76	22.64	0.91	0.31	0.00	0.31	3.20	3.29	6.44
				Standard normal variate and detrending, second-order polynominal	0.99	0.09	0.00	0.09	11.72	11.76	22.65	0.91	0.30	0.01	0.31	3.23	3.31	6.49
	Smoothing Savitzky-Golay derivative	3	Savitzky-Golay 2st derivative, first-order polynomial (within the command "Transform>Derivative>SG" in Unscrambler)	None	0.99	0.07	0.00	0.07	13.78	13.81	26.59	0.86	0.39	-0.01	0.39	2.12	2.57	5.05
				Standard normal variate	0.99	0.08	0.00	0.08	11.94	11.98	23.06	0.77	0.49	0.02	0.49	1.64	2.05	3.88
				Multiplicative scatter correction	0.99	0.11	0.00	0.11	9.37	9.42	18.14	0.72	0.53	0.03	0.53	1.54	1.90	3.56
				Standard normal variate and mean centering	0.99	0.08	0.00	0.08	11.94	11.98	23.06	0.77	0.49	0.01	0.49	1.61	2.04	3.86
				Standard normal variate and detrending, second-order polynominal	0.99	0.08	0.00	0.08	11.94	11.98	23.06	0.76	0.50	0.02	0.50	1.61	2.02	3.82
	Moving average	11	Raw data	None	0.96	0.21	0.00	0.21	4.73	4.83	9.30	0.94	0.25	0.00	0.25	4.01	4.10	7.24
				Standard normal variate	0.96	0.21	0.00	0.21	4.71	4.81	9.27	0.94	0.24	0.00	0.24	4.09	4.14	7.40
				Multiplicative scatter correction	0.96	0.20	0.00	0.20	4.92	5.02	9.67	0.94	0.24	0.00	0.24	4.15	4.18	7.61
				Standard normal variate and mean centering	0.96	0.21	0.00	0.21	4.71	4.81	9.27	0.94	0.24	0.00	0.24	4.13	4.18	7.47
				Standard normal variate and detrending, second-order polynominal	0.96	0.20	0.00	0.20	5.02	5.12	9.86	0.95	0.23	0.00	0.23	4.26	4.31	7.87
	Moving average	25	Raw data	None	0.96	0.21	0.00	0.21	4.67	4.78	9.20	0.94	0.25	0.00	0.25	3.97	4.05	7.16
				Standard normal variate	0.96	0.21	0.00	0.21	4.72	4.82	9.28	0.94	0.25	0.00	0.25	4.08	4.10	7.36
				Multiplicative scatter correction	0.96	0.20	0.00	0.20	4.84	4.94	9.51	0.94	0.25	0.00	0.25	4.04	4.07	7.38
				Standard normal variate and mean centering	0.96	0.21	0.00	0.21	4.72	4.82	9.28	0.94	0.25	0.00	0.25	4.06	4.08	7.32
				Standard normal variate and detrending, second-order polynominal	0.96	0.20	0.00	0.20	5.00	5.10	9.81	0.94	0.24	0.00	0.24	4.16	4.22	7.71
	Savitzky-Golay, 0-order polynomial (within the command	11	Raw data	None	0.96	0.21	0.00	0.21	4.72	4.83	9.29	0.94	0.24	0.00	0.24	4.04	4.12	7.29
				Standard normal variate	0.96	0.21	0.00	0.21	4.77	4.87	9.38	0.94	0.24	0.00	0.24	4.11	4.16	7.42
				Multiplicative scatter correction	0.96	0.20	0.00	0.20	4.91	5.02	9.66	0.94	0.24	0.00	0.24	4.12	4.16	7.57
				Standard normal variate and mean centering	0.96	0.21	0.00	0.21	4.77	4.87	9.38	0.94	0.24	0.00	0.25	4.08	4.11	7.34
				Standard normal variate and detrending, second-order polynominal	0.96	0.19	0.00	0.19	5.08	5.18	9.98	0.95	0.23	0.00	0.23	4.31	4.34	7.96
	Savitzky-Golay, 0-order polynomial (within the command	25	Raw data	None	0.96	0.21	0.00	0.21	4.67	4.78	9.21	0.94	0.25	0.00	0.25	3.99	4.07	7.21
				Standard normal variate	0.96	0.21	0.00	0.21	4.73	4.83	9.30	0.94	0.25	0.01	0.25	4.05	4.06	7.30
				Multiplicative scatter correction	0.96	0.20	0.00	0.20	4.85	4.95	9.53	0.94	0.24	0.00	0.25	4.08	4.12	7.47
				Standard normal variate and mean centering	0.96	0.21	0.00	0.21	4.73	4.83	9.30	0.94	0.24	0.01	0.24	4.15	4.19	7.53
				Standard normal variate and detrending, second-order polynominal	0.96	0.20	0.00	0.20	5.02	5.12	9.86	0.95	0.23	0.01	0.23	4.31	4.35	7.99
	Savitzky-Golay 1st derivative, first-order polynomial (within the command "Transform>Derivative>SG" in Unscrambler)			None	0.98	0.14	0.00	0.14	7.28	7.35	14.14	0.95	0.22	-0.01	0.22	4.50	4.57	8.83
				Standard normal variate	0.98	0.13	0.00	0.13	7.91	7.97	15.35	0.93	0.27	0.00	0.27	3.74	3.79	7.17
				Multiplicative scatter correction	0.98	0.13	0.00	0.13	7.95	8.02	15.44	0.93	0.26	0.00	0.26	3.75	3.82	7.10
		11		Standard normal variate and mean centering	0.98	0.13	0.00	0.13	7.91	7.97	15.35	0.93	0.26	0.00	0.27	3.73	3.81	7.22
				Standard normal variate and detrending, second-order polynominal	0.98	0.13	0.00	0.13	7.80	7.87	15.15	0.93	0.27	0.00	0.27	3.67	3.71	6.87
	Savitzky-Golay 2st derivative, second-order polynomial (within the command "Transform>Derivative>SG" in Unscrambler)			None	0.99	0.09	0.00	0.09	11.12	11.17	21.50	0.92	0.28	-0.01	0.28	3.50	3.56	6.90
				Standard normal variate	0.99	0.10	0.00	0.10	10.16	10.21	19.66	0.89	0.33	-0.01	0.33	2.97	3.06	5.85
				Multiplicative scatter correction	0.99	0.11	0.00	0.11	9.49	9.54	18.37	0.89	0.34	0.02	0.34	2.86	2.98	5.52
		11		Standard normal variate and mean centering	0.99	0.10	0.00	0.10	10.16	10.21	19.66	0.89	0.33	0.01	0.33	2.92	3.04	5.81
				Standard normal variate and detrending, second-order polynominal	0.99	0.10	0.00	0.10	10.14	10.19	19.62	0.89	0.33	0.01	0.33	2.90	3.03	5.80
	Savitzky-Golay, 1st derivative, first-order polynomial (within the			None	0.97	0.17	0.00	0.17	5.88	5.96	11.48	0.95	0.23	0.00	0.23	4.33	4.37	8.34
				Standard normal variate	0.97	0.17	0.00	0.17	5.98	6.07	11.68	0.94	0.24	0.00	0.24	4.09	4.18	8.04
				Multiplicative scatter correction	0.97	0.17	0.00	0.17	5.98	6.07	11.68	0.94	0.25	0.01	0.25	3.96	4.04	7.37
		25		Standard normal variate and mean centering	0.97	0.17	0.00	0.17	5.98	6.07	11.68	0.94	0.25	0.00	0.25	3.92	4.02	7.73
				Standard normal variate and detrending, second-order polynominal	0.97	0.17	0.00	0.17	5.99	6.07	11.69	0.94	0.24	0.00	0.24	4.09	4.15	7.94
	Savitzky-Golay, 2st derivative, second-order polynomial (within the			None	0.98	0.14	0.00	0.14	7.11	7.18	13.81	0.94	0.24	0.00	0.24	4.18	4.26	8.11
				Standard normal variate	0.98	0.13	0.00	0.13	7.49	7.56	14.55	0.92	0.28	-0.01	0.28	3.54	3.59	6.50
				Multiplicative scatter correction	0.98	0.13	0.00	0.13	7.45	7.52	14.48	0.93	0.27	0.00	0.27	3.73	3.77	6.89
		25		Standard normal variate and mean centering	0.98	0.13	0.00	0.13	7.49	7.56	14.55	0.92	0.28	0.00	0.28	3.61	3.64	6.60
				Standard normal variate and detrending, second-order polynominal	0.98	0.13	0.00	0.13	7.51	7.57	14.58	0.93	0.27	-0.01	0.27	3.68	3.73	6.75

	Smoothing	Search window	Filtering	Enhancing technique	Calibration							Validation						
					R2	RMSE	Bias	SEP	RPD pre	RPD lab	$RPIQ_{lab}$	R2	RMSE	Bias	SEP	RPD pre	RPD lab	$RPIQ_{lab}$
Ccarb [%] N=120	None	0	Raw data	None	0.90	0.22	0.00	0.22	3.06	3.22	3.95	0.87	0.26	0.00	0.26	2.67	2.77	3.40
				Standard normal variate	0.91	0.21	0.00	0.21	3.21	3.36	4.13	0.84	0.29	0.01	0.29	2.46	2.45	2.61
				Multiplicative scatter correction	0.91	0.21	0.00	0.21	3.27	3.42	4.20	0.83	0.32	0.00	0.32	2.39	2.26	2.31
				Standard normal variate and mean centering	0.91	0.21	0.00	0.21	3.21	3.36	4.13	0.84	0.29	0.01	0.29	2.46	2.45	2.61
				Standard normal variate and detrending, second-order polynominal	0.91	0.21	0.00	0.21	3.19	3.34	4.10	0.84	0.29	0.01	0.29	2.44	2.45	2.72
	Smoothing Savitzky-Golay derivative	3	Savitzky-Golay 1st derivative, first-order polynomial (within the command “Transform>Derivative>SG” in	None	0.98	0.10	0.00	0.10	7.46	7.52	9.24	0.86	0.27	0.00	0.27	2.63	2.65	3.24
				Standard normal variate	0.98	0.09	0.00	0.09	8.05	8.12	9.97	0.85	0.28	0.01	0.28	2.47	2.55	3.13
				Multiplicative scatter correction	0.98	0.09	0.00	0.09	7.88	7.94	9.76	0.85	0.28	0.00	0.28	2.51	2.57	3.13
				Standard normal variate and mean centering	0.98	0.09	0.00	0.09	8.05	8.12	9.97	0.86	0.27	0.01	0.27	2.59	2.68	3.29
				Standard normal variate and detrending, second-order polynominal	0.99	0.09	0.00	0.09	8.21	8.27	10.16	0.85	0.28	0.01	0.28	2.48	2.55	3.17
	Smoothing Savitzky-Golay derivative	3	Savitzky-Golay 2st derivative, first-order polynomial (within the command “Transform>Derivative>SG” in Unscrambler)	None	0.99	0.08	0.00	0.08	9.10	9.16	11.25	0.63	0.44	0.02	0.44	1.24	1.64	1.87
				Standard normal variate	0.99	0.07	0.00	0.07	9.65	9.70	11.92	0.59	0.46	-0.01	0.46	1.17	1.56	1.78
				Multiplicative scatter correction	0.99	0.08	0.00	0.08	9.46	9.51	11.69	0.64	0.43	-0.02	0.43	1.23	1.65	1.96
				Standard normal variate and mean centering	0.99	0.07	0.00	0.07	9.65	9.70	11.92	0.60	0.45	-0.01	0.45	1.18	1.59	1.82
				Standard normal variate and detrending, second-order polynominal	0.99	0.07	0.00	0.07	9.65	9.70	11.92	0.61	0.44	0.00	0.45	1.19	1.61	1.84
	Moving average	11	Raw data	None	0.90	0.23	0.00	0.23	3.00	3.17	3.89	0.87	0.26	0.00	0.26	2.65	2.75	2.47
				Standard normal variate	0.90	0.23	0.00	0.23	2.99	3.16	3.88	0.84	0.29	-0.01	0.29	2.42	2.46	2.31
				Multiplicative scatter correction	0.90	0.22	0.00	0.22	3.10	3.26	4.00	0.82	0.31	0.00	0.31	2.30	2.32	2.41
				Standard normal variate and mean centering	0.90	0.23	0.00	0.23	2.99	3.16	3.88	0.84	0.29	0.00	0.29	2.47	2.48	2.33
				Standard normal variate and detrending, second-order polynominal	0.90	0.23	0.00	0.23	2.97	3.14	3.85	0.82	0.31	0.01	0.31	2.34	2.34	2.40
	Moving average	25	Raw data	None	0.90	0.23	0.00	0.23	2.99	3.15	3.87	0.86	0.26	0.00	0.27	2.59	2.70	2.40
				Standard normal variate	0.90	0.22	0.00	0.22	3.05	3.21	3.94	0.83	0.30	0.01	0.30	2.35	2.37	2.43
				Multiplicative scatter correction	0.90	0.22	0.00	0.23	3.03	3.19	3.92	0.83	0.30	0.02	0.30	2.33	2.40	2.43
				Standard normal variate and mean centering	0.90	0.22	0.00	0.22	3.05	3.21	3.94	0.84	0.29	0.00	0.30	2.47	2.43	2.50
				Standard normal variate and detrending, second-order polynominal	0.90	0.23	0.00	0.23	2.99	3.16	3.88	0.85	0.28	0.00	0.29	2.50	2.52	2.74
	Savitzky-Golay, 0-order polynomial (within the command	11	Raw data	None	0.90	0.23	0.00	0.23	3.01	3.17	3.89	0.87	0.26	0.00	0.26	2.64	2.74	
				Standard normal variate	0.90	0.22	0.00	0.22	3.06	3.22	3.96	0.84	0.29	-0.01	0.29	2.47	2.45	2.58
				Multiplicative scatter correction	0.90	0.23	0.00	0.23	2.95	3.12	3.83	0.80	0.33	0.00	0.33	2.22	2.15	2.35
				Standard normal variate and mean centering	0.90	0.22	0.00	0.22	3.06	3.22	3.96	0.81	0.32	0.02	0.32	2.25	2.24	2.36
				Standard normal variate and detrending, second-order polynominal	0.90	0.22	0.00	0.22	3.06	3.22	3.95	0.84	0.29	0.00	0.29	2.44	2.45	2.69
	Savitzky-Golay, 0-order polynomial (within the command	25	Raw data	None	0.90	0.23	0.00	0.23	2.99	3.16	3.88	0.87	0.26	0.00	0.26	2.63	2.76	2.41
				Standard normal variate	0.90	0.22	0.00	0.23	3.02	3.18	3.91	0.82	0.31	-0.01	0.31	2.36	2.33	2.41
				Multiplicative scatter correction	0.90	0.23	0.00	0.23	3.00	3.17	3.89	0.83	0.30	0.01	0.30	2.31	2.37	2.39
				Standard normal variate and mean centering	0.90	0.22	0.00	0.23	3.02	3.18	3.91	0.82	0.31	0.00	0.31	2.34	2.31	2.39
				Standard normal variate and detrending, second-order polynominal	0.90	0.23	0.00	0.23	2.98	3.15	3.87	0.84	0.29	0.00	0.29	2.45	2.47	2.67
	Savitzky-Golay 1st derivative, first-order polynomial (within the command “Transform>Derivative>SG” in Unscrambler)			None	0.96	0.14	0.00	0.14	5.17	5.27	6.47	0.90	0.23	0.00	0.23	3.09	3.13	3.87
				Standard normal variate	0.98	0.11	0.00	0.11	6.30	6.38	7.83	0.89	0.24	0.01	0.24	2.94	2.93	3.81
				Multiplicative scatter correction	0.97	0.12	0.00	0.12	6.11	6.19	7.61	0.89	0.25	0.01	0.25	2.97	2.91	3.84
		11		Standard normal variate and mean centering	0.98	0.11	0.00	0.11	6.30	6.38	7.83	0.89	0.24	0.01	0.24	2.95	2.95	3.83
				Standard normal variate and detrending, second-order polynominal	0.98	0.11	0.00	0.11	6.27	6.35	7.80	0.89	0.24	0.01	0.24	2.95	2.95	3.83
	Savitzky-Golay 2st derivative, second-order polynomial (within the command “Transform>Derivative>SG” in Unscrambler)			None	0.98	0.09	0.00	0.09	7.79	7.85	9.64	0.86	0.27	0.00	0.27	2.60	2.66	3.22
				Standard normal variate	0.99	0.08	0.00	0.08	8.97	9.03	11.09	0.89	0.24	0.00	0.24	2.87	3.00	3.81
				Multiplicative scatter correction	0.99	0.08	0.00	0.08	8.83	8.89	10.92	0.88	0.25	0.01	0.25	2.78	2.85	3.65
		11		Standard normal variate and mean centering	0.99	0.08	0.00	0.08	8.97	9.03	11.09	0.89	0.23	0.01	0.23	2.95	3.08	3.92
				Standard normal variate and detrending, second-order polynominal	0.99	0.08	0.00	0.08	8.97	9.03	11.09	0.89	0.24	0.01	0.24	2.83	2.96	3.77
	Savitzky-Golay, 1st derivative, first-order polynomial (within the			None	0.95	0.16	0.00	0.17	4.23	4.35	5.34	0.90	0.23	0.01	0.23	3.10	3.14	3.55
				Standard normal variate	0.96	0.15	0.00	0.15	4.81	4.92	6.04	0.88	0.25	0.01	0.25	2.84	2.82	3.50
				Multiplicative scatter correction	0.95	0.16	0.00	0.16	4.32	4.44	5.45	0.87	0.26	0.01	0.26	2.78	2.71	3.35
		25		Standard normal variate and mean centering	0.96	0.15	0.00	0.15	4.81	4.92	6.04	0.88	0.25	0.01	0.25	2.86	2.82	3.51
				Standard normal variate and detrending, second-order polynominal	0.95	0.15	0.00	0.15	4.54	4.65	5.72	0.87	0.27	0.01	0.27	2.76	2.69	3.35
	Savitzky-Golay, 2st derivative, second-order polynomial (within the			None	0.97	0.13	0.00	0.13	5.39	5.49	6.74	0.91	0.22	0.00	0.22	3.25	3.30	4.20
				Standard normal variate	0.97	0.11	0.00	0.11	6.26	6.34	7.79	0.91	0.22	0.02	0.22	3.35	3.33	4.23
				Multiplicative scatter correction	0.97	0.12	0.00	0.12	5.99	6.08	7.47	0.89	0.24	0.00	0.24	3.03	3.02	3.73
		25		Standard normal variate and mean centering	0.97	0.11	0.00	0.11	6.26	6.34	7.79	0.92	0.21	0.02	0.21	3.43	3.42	4.34
				Standard normal variate and detrending, second-order polynominal	0.98	0.11	0.00	0.11	6.30	6.38	7.83	0.91	0.22	0.02	0.22	3.23	3.22	4.11

	Smoothing	Search window	Filtering	Enhancing technique	Calibration							Validation						
					R2	RMSE	Bias	SEP	RPD pre	RPD lab	$RPIQ_{lab}$	R2	RMSE	Bias	SEP	RPD pre	RPD lab	$RPIQ_{lab}$
C/N	None	0	Raw data	None	0.68	1.24	0.00	1.24	1.47	1.78	0.93	0.46	1.66	-0.04	1.67	1.15	1.32	0.69
				Standard normal variate	0.70	1.20	0.00	1.20	1.54	1.84	0.96	0.48	1.62	-0.04	1.63	1.17	1.36	1.09
N=120				Multiplicative scatter correction	0.73	1.13	0.00	1.14	1.66	1.94	1.01	0.46	1.67	0.00	1.67	1.15	1.32	1.10
				Standard normal variate and mean centering	0.70	1.20	0.00	1.20	1.54	1.84	0.96	0.48	1.62	-0.04	1.63	1.17	1.36	1.09
				Standard normal variate and detrending, second-order polynominal	0.72	1.17	0.00	1.17	1.59	1.88	0.98	0.46	1.67	0.00	1.68	1.14	1.32	0.90
	Smoothing Savitzky-Golay derivative	3	Savitzky-Golay 1st derivative, first-order polynomial (within the command “Transform>Derivative>SG” in	None	0.95	0.49	0.00	0.49	4.38	4.50	2.35	0.45	1.66	-0.02	1.67	1.11	1.32	0.98
				Standard normal variate	0.96	0.46	0.00	0.46	4.69	4.79	2.50	0.36	1.84	0.02	1.85	1.01	1.19	0.85
				Multiplicative scatter correction	0.96	0.45	0.00	0.46	4.72	4.83	2.52	0.45	1.66	-0.03	1.67	1.11	1.32	0.93
				Standard normal variate and mean centering	0.96	0.46	0.00	0.46	4.69	4.79	2.50	0.42	1.72	-0.03	1.73	1.07	1.28	0.91
				Standard normal variate and detrending, second-order polynominal	0.96	0.46	0.00	0.47	4.63	4.74	2.47	0.38	1.79	-0.01	1.80	1.04	1.23	0.87
	Smoothing Savitzky-Golay derivative	3	Savitzky-Golay 2st derivative, first-order polynomial (within the command “Transform>Derivative>SG” in Unscrambler)	None	0.98	0.27	0.00	0.28	7.95	8.01	4.18	0.32	1.85	-0.02	1.86	0.88	1.19	0.69
				Standard normal variate	0.98	0.34	0.00	0.34	6.34	6.42	3.35	0.26	1.96	-0.05	1.97	0.85	1.12	0.74
				Multiplicative scatter correction	0.97	0.38	0.00	0.38	5.75	5.84	3.05	0.27	1.96	-0.08	1.97	0.88	1.12	0.80
				Standard normal variate and mean centering	0.98	0.34	0.00	0.34	6.34	6.42	3.35	0.26	1.97	-0.02	1.98	0.86	1.11	0.74
				Standard normal variate and detrending, second-order polynominal	0.98	0.34	0.00	0.34	6.34	6.42	3.35	0.26	1.97	0.00	1.98	0.88	1.11	0.74
	Moving average	11	Raw data	None	0.67	1.26	0.00	1.26	1.43	1.75	0.91	0.43	1.69	-0.02	1.69	1.07	1.30	1.04
				Standard normal variate	0.70	1.20	0.00	1.20	1.54	1.84	0.96	0.47	1.63	0.04	1.63	1.14	1.35	1.15
				Multiplicative scatter correction	0.71	1.18	0.00	1.18	1.57	1.87	0.97	0.45	1.68	0.01	1.68	1.14	1.31	1.14
				Standard normal variate and mean centering	0.70	1.20	0.00	1.20	1.54	1.84	0.96	0.53	1.52	0.03	1.53	1.19	1.44	1.23
				Standard normal variate and detrending, second-order polynominal	0.72	1.17	0.00	1.17	1.59	1.88	0.98	0.43	1.74	-0.05	1.74	1.13	1.27	1.04
	Moving average	25	Raw data	None	0.66	1.27	0.00	1.27	1.41	1.73	0.90	0.45	1.68	-0.02	1.68	1.11	1.31	1.05
				Standard normal variate	0.68	1.23	0.00	1.24	1.47	1.78	0.93	0.47	1.63	0.04	1.64	1.12	1.35	1.16
				Multiplicative scatter correction	0.70	1.20	0.00	1.20	1.54	1.84	0.96	0.45	1.66	0.03	1.67	1.11	1.32	1.18
				Standard normal variate and mean centering	0.68	1.23	0.00	1.24	1.47	1.78	0.93	0.50	1.57	0.01	1.57	1.15	1.40	1.20
				Standard normal variate and detrending, second-order polynominal	0.69	1.22	0.00	1.22	1.50	1.81	0.94	0.44	1.70	-0.03	1.71	1.13	1.29	0.96
	Savitzky-Golay, 0-order polynomial (within the command	11	Raw data	None	0.67	1.25	0.00	1.26	1.43	1.75	0.91	0.46	1.65	0.02	1.66	1.11	1.33	
				Standard normal variate	0.69	1.22	0.00	1.23	1.49	1.80	0.94	0.50	1.56	0.03	1.57	1.15	1.40	1.16
				Multiplicative scatter correction	0.71	1.18	0.00	1.18	1.57	1.87	0.97	0.47	1.65	0.02	1.66	1.16	1.33	1.16
				Standard normal variate and mean centering	0.69	1.22	0.00	1.23	1.49	1.80	0.94	0.50	1.56	0.05	1.57	1.12	1.41	1.16
				Standard normal variate and detrending, second-order polynominal	0.70	1.21	0.00	1.21	1.52	1.82	0.95	0.43	1.72	0.03	1.73	1.12	1.28	0.92
	Savitzky-Golay, 0-order polynomial (within the command	25	Raw data	None	0.66	1.27	0.00	1.27	1.41	1.73	0.90	0.44	1.67	-0.01	1.68	1.10	1.31	1.04
				Standard normal variate	0.68	1.23	0.00	1.24	1.47	1.78	0.93	0.50	1.58	0.03	1.58	1.15	1.39	1.15
				Multiplicative scatter correction	0.70	1.20	0.00	1.20	1.54	1.84	0.96	0.46	1.64	-0.03	1.65	1.13	1.34	1.18
				Standard normal variate and mean centering	0.68	1.23	0.00	1.24	1.47	1.78	0.93	0.48	1.61	0.01	1.62	1.15	1.36	1.12
				Standard normal variate and detrending, second-order polynominal	0.69	1.22	0.00	1.22	1.50	1.81	0.94	0.43	1.70	0.05	1.71	1.08	1.29	0.96
	Savitzky-Golay 1st derivative, first-order polynomial (within the command “Transform>Derivative>SG” in Unscrambler)			None	0.86	0.82	0.00	0.83	2.47	2.67	1.39	0.49	1.62	-0.04	1.62	1.23	1.36	1.11
				Standard normal variate	0.89	0.71	0.00	0.71	2.92	3.09	1.61	0.54	1.53	0.00	1.54	1.29	1.43	1.12
				Multiplicative scatter correction	0.89	0.71	0.00	0.71	2.93	3.10	1.62	0.56	1.48	-0.04	1.48	1.32	1.49	1.17
		11		Standard normal variate and mean centering	0.89	0.71	0.00	0.71	2.92	3.09	1.61	0.54	1.53	-0.05	1.53	1.30	1.44	1.13
				Standard normal variate and detrending, second-order polynominal	0.89	0.72	0.00	0.72	2.90	3.07	1.60	0.50	1.60	-0.04	1.60	1.22	1.38	1.17
	Savitzky-Golay 2st derivative, second-order polynomial (within the command “Transform>Derivative>SG” in Unscrambler)			None	0.95	0.47	0.00	0.48	4.52	4.63	2.42	0.40	1.79	-0.04	1.80	1.10	1.23	0.94
				Standard normal variate	0.95	0.47	0.00	0.48	4.52	4.63	2.42	0.34	1.93	-0.06	1.93	1.06	1.14	0.72
				Multiplicative scatter correction	0.95	0.49	0.00	0.50	4.34	4.45	2.32	0.36	1.89	0.00	1.90	1.09	1.16	0.66
		11		Standard normal variate and mean centering	0.95	0.47	0.00	0.48	4.52	4.63	2.42	0.35	1.94	-0.10	1.94	1.07	1.13	0.72
				Standard normal variate and detrending, second-order polynominal	0.95	0.48	0.00	0.48	4.51	4.62	2.41	0.35	1.91	0.00	1.92	1.06	1.15	0.73
	Savitzky-Golay, 1st derivative, first-order polynomial (within the			None	0.76	1.07	0.00	1.07	1.79	2.05	1.07	0.48	1.62	-0.04	1.63	1.16	1.36	1.38
				Standard normal variate	0.79	0.99	0.00	1.00	1.97	2.21	1.16	0.43	1.73	-0.05	1.73	1.13	1.27	1.07
				Multiplicative scatter correction	0.80	0.98	0.00	0.99	2.00	2.23	1.17	0.49	1.62	-0.04	1.63	1.22	1.36	1.20
		25		Standard normal variate and mean centering	0.79	0.99	0.00	1.00	1.97	2.21	1.16	0.45	1.70	-0.06	1.71	1.17	1.29	1.08
				Standard normal variate and detrending, second-order polynominal	0.79	1.00	0.00	1.01	1.94	2.19	1.14	0.41	1.76	-0.05	1.76	1.08	1.25	1.15
	Savitzky-Golay, 2st derivative, second-order polynomial (within the			None	0.87	0.79	0.00	0.79	2.60	2.79	1.46	0.52	1.55	-0.01	1.56	1.23	1.41	1.17
				Standard normal variate	0.88	0.77	0.00	0.77	2.68	2.86	1.49	0.41	1.77	0.01	1.78	1.12	1.24	0.97
				Multiplicative scatter correction	0.88	0.76	0.00	0.76	2.72	2.90	1.51	0.49	1.62	0.04	1.62	1.19	1.36	1.08
		25		Standard normal variate and mean centering	0.88	0.77	0.00	0.77	2.68	2.86	1.49	0.39	1.88	0.02	1.89	1.14	1.17	0.91
				Standard normal variate and detrending, second-order polynominal	0.88	0.77	0.00	0.77	2.69	2.87	1.50	0.43	1.73	-0.03	1.74	1.13	1.27	0.99
